# Metformin, Macrophage Dysfunction and Atherosclerosis

**DOI:** 10.3389/fimmu.2021.682853

**Published:** 2021-06-07

**Authors:** Xiaojun Feng, Wenxu Chen, Xiayun Ni, Peter J. Little, Suowen Xu, Liqin Tang, Jianping Weng

**Affiliations:** ^1^ Department of Pharmacy, the First Affiliated Hospital of University of USTC, Division of Life Sciences and Medicine, University of Science and Technology of China (USTC), Hefei, China; ^2^ Sunshine Coast Health Institute, University of the Sunshine Coast, Birtinya, QLD, Australia; ^3^ School of Pharmacy, Pharmacy Australia Centre of Excellence, The University of Queensland, Woolloongabba, QLD, Australia; ^4^ Department of Endocrinology, The First Affiliated Hospital of USTC, Division of Life Sciences and Medicine, University of Science and Technology of China( USTC), Hefei, China

**Keywords:** metformin, atherosclerosis, macrophage, NETs, combination medication

## Abstract

Metformin is one of the most widely prescribed hypoglycemic drugs and has the potential to treat many diseases. More and more evidence shows that metformin can regulate the function of macrophages in atherosclerosis, including reducing the differentiation of monocytes and inhibiting the inflammation, oxidative stress, polarization, foam cell formation and apoptosis of macrophages. The mechanisms by which metformin regulates the function of macrophages include AMPK, AMPK independent targets, NF-κB, ABCG5/8, Sirt1, FOXO1/FABP4 and HMGB1. On the basis of summarizing these studies, we further discussed the future research directions of metformin: single-cell RNA sequencing, neutrophil extracellular traps (NETs), epigenetic modification, and metformin-based combination drugs. In short, macrophages play an important role in a variety of diseases, and improving macrophage dysfunction may be an important mechanism for metformin to expand its pleiotropic pharmacological profile. In addition, the combination of metformin with other drugs that improve the function of macrophages (such as SGLT2 inhibitors, statins and IL-1β inhibitors/monoclonal antibodies) may further enhance the pleiotropic therapeutic potential of metformin in conditions such as atherosclerosis, obesity, cancer, dementia and aging.

## Introduction

Type 2 diabetes mellitus (T2DM) is a global epidemic. T2DM is highly prevalent in Asia, with increased prevalence in India and China (1). Although genetic factors determine the susceptibility of individuals to T2DM to a considerable extent, a sedentary lifestyle and obesogenic diet are important predisposing factors for the current global epidemic (1, 2). Studies show lifestyle changes can prevent T2DM, including maintaining a healthy weight and diet, maintaining physical activity, not smoking and drinking moderately (1–3). Most T2DM patients have at least one morbidity, termed a complication (including cardiovascular, renal, retinal and neurological complications) (1–3). Accelerated atherosclerosis (AS) is the main cause of the high incidence of cardiovascular (CV) complications and premature death in patients with diabetes (4, 5). Atherosclerosis, depending on the vascular bed affected, can result in coronary artery disease (CAD), stroke, and peripheral arterial disease (6).

The oxidation of low-density lipoprotein cholesterol (LDL-c) is considered to be one of the most important of the plethora of factors associated with the occurrence and development of AS (7, 8). Lowering LDL plasma levels is a first line clinical treatment to decrease the risk profile of patients for cardiovascular and cerebrovascular diseases (7, 9). In the initial stage of the development of atherosclerotic lesions, plasma lipoproteins trapped by modified proteoglycans accumulate in the subendothelial space, which can cause endothelial cell (EC) and platelet activation (8–12). Circulating monocytes adhere to the activated endothelium, penetrate the endothelium and differentiate into pro-inflammatory macrophages, which form foam cells by increasing the uptake of oxLDL. This aggravated inflammation expands as foam cells spread over the growing atheroma (9, 10). The process of AS is accelerated by numerous factors, such as the generation of ROS, as well as the release of inflammatory chemokines and cytokines (9, 13). Regardless of whether or not there is diabetes present, monocytes and macrophages exhibit a very important role in all stages of the development of AS. The diabetic environment further changes the phenotype of monocytes and macrophages, such as promoting the recruitment of monocytes to the lesion, further activating inflammation, increasing the accumulation and metabolism of lipids in macrophages, and increasing the death of macrophages (14–16). Macrophage pathobiology has become a new potential target for anti-atherosclerosis therapy (14, 17).

Metformin was prepared and synthesized in 1922, and the hypoglycemic effect of metformin was confirmed through clinical trials in 1957 (18). In 1995, metformin was approved for the therapy of T2DM in the United States (18). The discovery of metformin opened a new page in the emerging struggle between humans and diabetes (18, 19). With the publication of a number of large-scale clinical studies with positive outcomes, metformin has been widely accepted as the first-line therapy for T2DM (20). Metformin mainly activates AMPK, thereby reducing hepatic glucose output (by inhibiting hepatic gluconeogenesis) and increasing glucose utilization in peripheral tissues (by increasing insulin receptor sensitivity and improving insulin resistance) (20, 21). In addition, the hypoglycemic mechanism of metformin also involves promoting the increase of glucagon-like peptide-1 (GLP-1) release, regulating the intestinal flora and promoting the excretion of blood glucose from the intestine (21). Metformin is a cardioprotective and is a hypoglycemic drug with clear evidence of cardiovascular benefits (22, 23). Mounting evidence have shown that long-term treatment of metformin is related to a significantly reduced risk of cardiovascular disease (CVD) in pre-T2DM patients and T2DM patients and even in some T1DM patients (**Table 1**). Metformin is being explored for new functions and pharmacological mechanisms.

**Table 1:** Clinical studies of metformin in diabetic patients with cardiovascular risk.

Subjects	Grouping	Results	Conclusion
T1DM who are at higher risk of CVD.	Metformin (1 g twice a day) + insulin (n=219) Placebo + insulin (n=219)	↓the maximum cIMT ↓body weight and LDL cholesterol ↑eGFR	Metformin may exhibit a greater effect in CVD risk management in patients with T1DM who are at higher risk of CVD (24).
Children with T1DM.	Metformin (1 g twice a day) + insulin (n=45) Placebo + insulin (n=45)	↑GTN ↓insulin dose ↓HbA1c	Metformin can improve HbA1c, the function of vascular smooth muscle and reduce insulin doses in children with T1DM (25).
T2DM	Metformin users (n = 7457) Non-users (n = 12 234)	↓mortality rates (HR=0.76, 0.65-0.89; P<0.001) ↓mortality rates of patients with a history of congestive heart failure (HR=0.69, 0.54-0.90; P=0.006).	Metformin treatment, as a means of secondary prevention, can reduce the mortality of diabetic patients (26).
Patients with T2DM and renal insufficiency.	Metformin users (n = 67749), Sulfonylureas (n = 28976) Weighted cohort metformin (n=24679), sulfonylureas (n=24799)	↓MACE rates (adjusted HR=0.80, 95% CI, 0.75-0.86)	In diabetic patients with decreased renal function treated with monotherapy, metformin treatment reduced the risk of MACE compared with sulfonylureas (27).
T2DM	Metformin group (n=20) (500 mg twice a day) Gliclazide group (n=20) Pioglitazone group (n=20)	↓LDL(3) mass and the LDL(3)-to-LDL ratio ↑HDL(2)-to -HDL(3) ratio	Compared with gliclazide, the content of HDL and LDL subgroups in the pioglitazone or metformin group changed favorably.Such changes may be related to reducing the risk of AS (28).
Patients with IGT	1645 pairs of matched samples (534 LSM, 558 metformin(850 mg twice daily) and 553 placebo).	LSM: ↑large HDL, ↓small HDL, small and dense LDL as well as large and buoyant VLDL Metformin: modestly ↑small and large HDL, ↓small and dense LDL	LSM or metformin treatment has a good effect on lipoprotein subcomponents, which may delay the development of diabetes and AS (29).
Pre-DM	Placebo (n=1082) Metformin (850 mg twice daily) (n=1073) Lifestyle (n=1079)	↓CAC severity and presence of men (CAC presence, 75% *vs.* 84%, CAC severity, 39.5 *vs.* 66.9 agatston units)	Metformin can prevent CAD in men with prediabetes and early diabetes (30).
HIV patients with MetS	No LSM (n=11), LSM (n=11), No LSM + metformin (n=13), LSM +metformin (n=15) (850 mg twice a day)	↓CAC improved HOMA-IR LSM had no obvious effect on CAC progression	These results show that metformin can prevent plaque progression in HIV-infected MetS patients (31).
First-degree relatives of T2DM patients with MetS	Placebo (n = 15) metformin (n = 16) (850 mg twice a day)	↓body weight, BMI, FPG and systolic blood pressure Improved blood lipids and endothelium-dependent FBF.	Metformin can improve the vascular endothelial function of first-degree relatives with MetS in patients with T2DM, regardless of the known hypoglycemic effect (32).
Patients with stable angina pectoris and NOCS diabetes.	86 normal blood glucose (NG) subjects, 86 pre-DM subjects and 86 metformin-treated pre-diabetes (pre-DM +metformin) subjects (850 mg twice a day)	↓percentage of endothelial LAD dysfunction ↓MACE (predicted by a multivariable logistic regression model)	Metformin treatment can decrease the high risk of MACE in pre-DM patients *via* improving coronary endothelial dysfunction (33).
Patients with CAD, IR and/or pre-DM.	Placebo (n=34) Metformin (n=34) (2 g once a day)	↓LVMI ↓LVM, office systolic blood pressure, subcutaneous adipose tissue, body weight, and thiobarbituric acid reactive substance concentrations (oxidative stress biomarkers)	Metformin treatment obviously decreased LVMI, LVM, local systolic blood pressure, and body weight. Large trials of cardiovascular results require definitive evidence to prove the cardioprotective effects of metformin (34).

In this table, we describe the role of metformin in clinical research on atherosclerotic diseases, including subjects, grouping, results and conclusions. ↑Represents increase or activation. ↓Represents to reducion or inhibition. The corresponding abbreviations are as follows: AS, atherosclerosis; BMI, body mass index; CAC, coronary artery calcification; cIMT, carotid intima-media thickness; CVD, cardiovascular disease; DM, diabetes mellitus; eGFR, estimated glomerular filtration rate; FBF, forearm blood flow; FPG, fasting blood glucose; GTN, glyceryl trinitrate-mediated dilatation; HIV, The human immunodeficiency virus; HDL, high-density lipoprotein; HOMA-IR, homeostatic model of assessment-insulin resistance; IGT, impaired glucose tolerance; IR, insulin resistance; LAD, left anterior descending coronary artery; LDL, low-density lipoprotein; LSM, lifestyle modification; LVH, left ventricular hypertrophy; LVM, left ventricular mass; LVMI, left ventricular mass indexed to height; MACE, major adverse cardiac events; MetS, metabolic syndrome; NG, normal blood glucose; NOCS, non-obstructive coronary artery stenosis; pre-DM, pre-diabetes; T1DM, type 1 diabetes;T2DM, type 2 diabetes.

The results of various preclinical studies and clinical trials have shown that metformin plays a protective role in CVD (**Tables 1** and **2**) (35, 36). Metformin exhibits a protective effect in all aspects of AS, including ECs dysfunction (19, 37), vascular smooth muscle cells (SMCs) proliferation and migration (38, 39), monocyte/macrophage differentiation (40), macrophage mediated inflammation (41), and foam cell formation (42). In addition, metformin improves vascular remodeling caused by pulmonary hypertension (43). Macrophages play an important role in T2DM and its complications (AS), and numerous studies have shown that metformin has a preventative role in the development of AS and in the regulation of the function of macrophages. We have analyzed and summarized the research about metformin in the modulation of macrophage function in AS: including roles in inflammation, polarization, oxidative stress, foam cell formation and apoptosis, and considered the relevant mechanisms of action. Finally, we looked forward to the future research on metformin in macrophage pathobiology, including non-AMPK-dependent mechanisms, epigenetic modification, single-cell sequencing, neutrophil extracellular traps (NETs), and actions of metformin when combined with other relevant drugs.

We searched the Pubmed and Google databases for the following information: first, we searched all public publications about metformin and macrophages, with the keyword “metformin and macrophages.” Second, we searched for other related publications, including the function of macrophages and neutrophils in AS, the effect of metformin on AS, non-coding RNA.

## Clinical Studies of Metformin in the Treatment of AS

Due to its high morbidity and mortality, CAD represents a major global health and economic burden (7, 44). Therefore, early recognition, diagnosis and timely treatment of risk factors is an essential component of patient care (35). Diabetes is an important independent risk factor for the occurrence of CAD (45). Metformin, as the most widely used prescription hypoglycemic agent, has been demonstrated to reduce CV events in patients with diabetes in a number of clinical studies (35, 36).

For example, Petrie, JR et al. (24) conducted a double-blind, placebo-controlled trial (REMOVAL) to investigate whether or not metformin (combined with insulin therapy) reduces AS in patients with T1DM who are at higher risk of CVD. In 23 hospital diabetes clinics in five countries (United Kingdom, Netherlands, Canada, Australia, and Denmark), subjects with age ≥ 40 years old, T1DM for at least 5 years and at least 10 specific CV risk factors were randomized to receive either placebo or metformin (1 g twice a day). Among the 428 randomly assigned subjects, there were 219 subjects in the placebo group and 209 subjects in the metformin group. Although the average common carotid intima-media thickness (cIMT), a surrogate measure of AS, progression of the metformin treatment group was not decreased, the maximum cIMT of the metformin group was decreased. In the metformin treated group, the average insulin requirement within 3 years did not decrease significantly, but body weight and LDL-c decreased, and estimated glomerular filtration rate (eGFR) increased. These results indicate that metformin may exhibit a greater effect in cardiovascular risk management (24). Children with T1DM have vascular dysfunction before AS. Early intervention is needed to prevent CVD. A 12-month randomized controlled double-blind trial involving 90 participants found that metformin (1 g twice a day) can ameliorate SMCs function and HbA1c in children with T1DM, and reduce insulin doses (25).

To assess whether metformin therapy affects the mortality caused by atherosclerotic thrombosis in patients with diabetes, a study was conducted which included 19,691 diabetic patients with AS in the Reduction of AS Continuing Health (REACH) registration between 2003 and 2004. They received or did not receive metformin treatment, and the 2-year mortality rate in these two groups was analyzed. The results showed that the mortality rates of the metformin group and non-metformin group were 6.3% and 9.8%, respectively, and the adjusted hazard ratio (HR) was 0.76 (0.65-0.89; P<0.001). The correlation of lower mortality among the subgroups was consistent. Patients with a history of congestive heart failure benefit more, with an HR of 0.69 (0.54-0.90; P=0.006). These results show that metformin may be used as a secondary prevention method to reduce the mortality of diabetic patients (26). Roumie, CL et al. studied the cardiovascular clinical outcome of metformin in patients with T2DM and renal insufficiency. Including new users of metformin or sulfonylureas, follow-up starts with a lowered renal function threshold lasting until the occurrence of major adverse cardiac events (MACE) (including myocardial infarction, heart failure, transient ischemic attack (TIA), ischemic or hemorrhagic stroke and CVD death), treatment changes, loss of follow-up, death, or the end of the study. The results showed that the number of patients who used metformin or sulfonylureas alone for a long time was 67749 and 28976, respectively; there were 24679 metformin and 24799 sulfonylurea users in the weighted cohort. During the follow-up period (median, 1.0 year for metformin and 1.2 years for sulfonylurea), there were 1394 events in sulfonylurea users (29.2 per thousand person-years) and 1048 MACE events in metformin users (23.0 per thousand person-years). Compared with sulfonylureas, the MACE adjusted HR of metformin is 0.80 (95% CI, 0.75-0.86). This study shows that compared with sulfonylureas, in patients with diabetes with renal insufficiency receiving monotherapy, metformin treatment may be associated with a lower risk of MACE (27).

Lawrence, JM et al. compared the effects of oral hypoglycemic drugs on lipoprotein sub-components in T2DM subjects. Sixty overweight T2DM subjects who did not receive lipid-lowering treatment were randomly assigned to metformin, pioglitazone or gliclazide after three months of diet run-in with adjustment of the drug dosage to optimize blood glucose control, and continuing treatment for 3 months. Compared with gliclazide, the content of high-density lipoprotein (HDL) and LDL subgroups in the pioglitazone or metformin group changed favorably (including the increase in HDL (2)-to -HDL (3) ratio, and the decrease in LDL (3) mass and the LDL (3)-to-LDL ratio) (28). Such changes may be related to reducing the risk of AS (28). Similarly, a randomized placebo-controlled clinical trial evaluated the effects of lifestyle modification (LSM) (a low-fat diet that reduces body weight by 7%) or metformin (850 mg twice a day) on patients with impaired glucose tolerance (IGT). LSM increased large HDL and decreased small HDL, small and dense LDL as well as large and buoyant Very Low Density Lipoproteins (VLDL). Metformin modestly raised small and large HDL as well as decreasing small and dense LDL. Metformin modestly raised small and large HDL and decreased small and dense LDL. Thus, metformin treatment has beneficial effects on lipoprotein subcomponent, but LSM may be more effective. Both interventions may delay the development and progression of AS (29).

Patients with prediabetes (pre-DM) are at a higher risk of CAD and may require prevention interventions to decrease the risk of CAD. The Diabetes Prevention Program Outcome Study (DPPOS) and the Diabetes Prevention Program (DPP) studied 3234 subjects with pre-DM. After an average follow-up time of 14 years, 2029 participants were assessed for AS through coronary artery calcium (CAC) measurements. Men in the metformin group had lower CAC severity and presence compared with the placebo group but no effect of metformin was observed in women. Metformin (850 mg twice daily) can prevent CAD in men with pre-DM and early diabetes (30). Human immunodeficiency virus (HIV) patients are prone to metabolic abnormalities (such as DM, obesity, hyperlipidemia, and hypertension), which can induce CAC. In 50 subjects with metabolic syndrome (MetS) infected with HIV, the effect of LSM and/or metformin (850 mg twice a day) treatment on the parameters of the MetS was studied. Subjects receiving metformin therapy for more than 1 year showed a significant reduction in CAC progression, while LSM had no effect on CAC progression. These results show that metformin therapy can prevent plaque development in HIV-infected MetS patients (31).

An early feature of AS in patients with T2DM is endothelial dysfunction. In this regard, thirty-one volunteers who were first-degree relatives of patients with T2DM, and had normal glucose tolerance and MetS were recruited. The volunteers were randomly assigned to a metformin (850 mg twice a day) group (n = 16) or a control group (n = 15). The weight, BMI, fasting blood glucose (FPG) and systolic blood pressure (BP) of the metformin group decreased, and the blood lipid profiles also improved. The measurement of the endothelium-dependent forearm blood flow (FBF) response also improved. These results indicate that metformin can improve the vascular endothelial responsiveness of first-degree relatives of T2DM patients with MetS (32). Similarly, the 258 stable angina patients matched by propensity scores included 86 normal blood glucose (NG) subjects, 86 pre-DM subjects and 86 metformin-treated pre-diabetes (Met+pre-DM) subjects. At the 24th month of follow-up, MACE of NG subjects and Met+pre-DM subjects were lower than those of pre-DM subjects. In addition, the percentage of left anterior descending coronary artery (LAD) endothelial dysfunction in NG subjects and Met+pre-DM subjects was also lower than that in pre-DM subjects. These findings show that metformin therapy can decrease the high risk of MACE in patients with pre-DM *via* improving coronary endothelial function (33). In addition, metformin has a beneficial effect on the regression of left ventricular hypertrophy (LVH) in patients with CAD, insulin resistance (IR) and/or prediabetes. The study conducted by Mohan, M et al. randomly assigned 68 subjects without diabetes but with CAD, IR and/or prediabetes to receive placebo or metformin (2 g once a day) for 12 months. The findings were that metformin therapy decreased weight, left ventricular mass (LVM), left ventricular mass indexed to height (LVMI), and systolic BP (34).

## Metformin in Regulating Macrophage Dysfunction

### Inflammation

**Figure 1:**
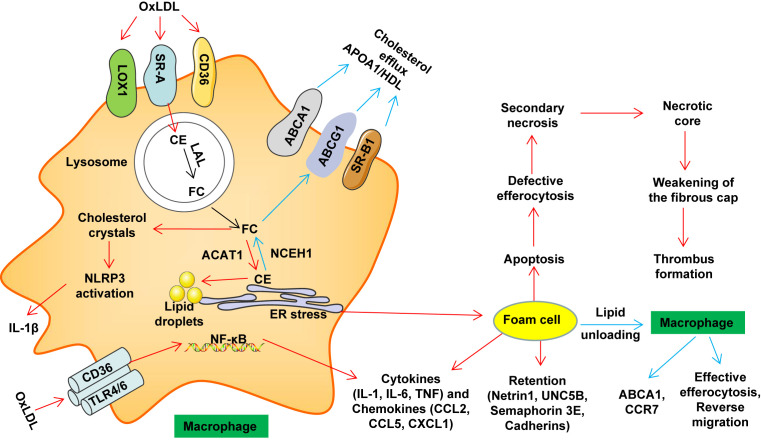
Macrophage lipid metabolism disorder and thrombosis. Macrophages phagocytose accumulated LDL and modified LDL (such as oxLDL) through multiple cell membrane scavenger receptors such as LOX1, SR-A and CD36. In the lysosome, cholesterol ester (CE) in LDL particles is degraded to free cholesterol (FC) and free fatty acids by lysosomal acid lipase (LAL). In the endoplasmic reticulum (ER), FC is re-esterified by acetyl-coenzyme A: cholesterol acetyltransferase 1 (ACAT1), which contributes to CE accumulation and lipid droplet formation. CE is hydrolyzed by neutral cholesterol ester hydrolase 1 (NCEH1) to release FC, and is further transported to the outside of the cell through cholesterol efflux transporters, ABCA1, ABCG1 and SR-B1, thereby maintaining cholesterol metabolism homeostasis. Under pathological conditions (such as AS), excessive accumulation of FC induces the formation of cholesterol crystals in the lysosome, which activates the NLRP3 inflammasome, induces ER stress, and ultimately leads to the formation of foam cells. OxLDL can also activate the NF-κB signaling pathway through the CD36–TLR4/TLR6 trimer. Foam cells secrete macrophage chemotaxis retention factors (including netrin 1 and its receptor UNC5B, cadherins and semaphorin 3E), pro-inflammatory cytokines (such as IL-1, IL-6 and TNF) and chemokines (such as CCL2, CCL5 and CXCL1) amplify the inflammatory response. Over time, foam cells undergo apoptosis. When these apoptotic cells cannot be effectively cleared by macrophages in advanced disease (defective erythrocytosis), secondary necrosis will result. Further development will promote the formation of necrotic cores. At the same time, the living macrophages in the late plaque secrete cytokines, proteases and procoagulant thrombosis factors, as well as VSMCs death and protease degradation of the extracellular matrix. These effects weaken the stability of the fibrous cap (easy to rupture). The rupture leads to exposure of the thrombogenic substances in these lesions, which eventually causes platelet aggregation and thrombosis, resulting in MACE events. ABCA1, ATP-binding cassette transporter A1; ABCG1, ATP-binding cassette transporter G1; CCL, C−C motif chemokine; CCR, C-C chemokine receptor; CD, cluster of differentiation; ER, endoplasmic reticulum; HDL, high-density lipoprotein; IL, Interleukin; LOX1, lectin-like oxidized LDL receptor 1; APOA1, lipid-poor apolipoprotein A1; NF-κB, nuclear factor kappa B; NLRP3, NOD-like receptor family pyrin domain containing 3; oxLDL, oxidized low-density lipoprotein; SRA, scavenger receptor class A; TLR, Toll-like receptor; SR-B1, scavenger receptor class B type 1.

Chronic inflammation or defective inflammation resolution plays a key role in the occurrence and development of AS (46). Macrophages phagocytose modified LDL (such as oxLDL) through multiple cell membrane scavenger receptors such as lectin-like oxidized LDL receptor-1 (LOX-1), scavenger receptor class A (SRA) and CD36 (16). In the lysosome, cholesterol ester (CE) in LDL particles is degraded to free cholesterol (FC) and free fatty acids by lysosomal acid lipase (LAL) (16, 47, 48). FC is re-esterified in the endoplasmic reticulum (ER) which contributes to CE accumulation and lipid droplet formation (16, 49). CE is hydrolyzed to release FC, and is further transported to the outside of the cell through cholesterol efflux transporters, ABCA1, ABCG1 and scavenger receptor class B type 1 (SR-B1); these processes maintain cholesterol metabolism homeostasis (16, 47). During the development and progression of AS, excessive accumulation of FC induces the formation of cholesterol crystals in the lysosome, which activates the nucleotide-binding oligomerization domain receptor, pyrin domain containing (NLRP)3 inflammasome, induces ER stress, and ultimately leads to the formation of foam cells (49). OxLDL can also activate the NF-κB signaling pathway through the CD36–Toll-like receptor (TLR)4/TLR6 trimer (49, 50). Foam cells secrete macrophage chemotaxis retention factors, causing pro-inflammatory cytokines and chemokines to amplify the inflammatory response (49, 50) (**Figure 1**). Excessive inflammation may induce plaque rupture and increase the risk of coronary thrombosis (51). Currently, evidence indicates that cytokines (such as IL-1β, IL-17 and TNF) are targets for reducing the progression of CVD at least in some cases (52).

Cytokines and chemokines exhibit a very important effect in inflammation, and some of them are therapeutic targets for attenuating chronic inflammatory diseases (53). Metformin may be beneficial for macrovascular complications of diabetes, as a complement to its hypoglycemic effect (54). In large-scale treatment of newly diagnosed diabetic patients, the difference in systemic inflammatory markers, neutrophil-lymphocyte ratios after treatment with sulfonylurea or metformin monotherapy was observed. Compared to sulfonylurea therapy, metformin decreased the mean log-transformed neutrophil to lymphocyte ratio by 0.09 U after 8 to 16 months. Following these findings in a non-diabetic heart failure trial (registration: NCT00473876), metformin inhibited blood cytokines, including the C-C motif chemokine 11 (CCL11). These findings reveal that metformin has anti-inflammatory effects in both diabetic and non-diabetic patients, a finding which may accelerate the study of the role of metformin in CVD in non-diabetic patients (55). Similarly, in the mouse macrophage cell line RAW264.7, in macrophages treated with lipopolysaccharide (LPS), metformin inhibited LPS-stimulated chemokine expression (including CCL2, CXCL10 and CXCL11) by activating AMPK and inhibiting the phosphorylation of I-κBα and p65 (53). A similar study showed that metformin can attenuate LPS-stimulated acute lung injury (ALI) capillary damage by activating AMPKα1, including reducing inflammatory cytokine release, neutrophil and macrophage infiltration, and reducing myeloperoxidase activity (56). Acute phase serum amyloid A (ASAA) is a pro-inflammatory adipokine which is up-regulated in obese and IR patients. Polycystic ovary syndrome (PCOS) is one of the most common metabolic disorders in premenopausal women, and is related to inflammation and AS. ASAA in serum and adipose tissue (AT) is up-regulated in women with PCOS. Metformin therapy can reduce blood ASAA in these women (57). In addition, in the LPS-induced mouse acute respiratory distress syndrome (ARDS) model, metformin reduced LPS-induced lung injury and inflammatory factor expression and mortality in mice. In cultured alveolar macrophages (NR8383), metformin inhibits the activation of mitogen-activated protein kinase (MAPK) (including p38 and ERK) signaling pathways and increases the expression of SIRT1 by decreasing mir-138-5p expression, which may be the mechanism by which metformin inhibits ARDS (58).

Inflammation is closely related to the progression of T2DM and AS (17, 59, 60). Metformin dose-dependently reduces IL-1β-stimulated production of IL-6 and IL-8 in human ECs, macrophages (Mphis) and SMCs. Mechanistic studies have indicated that metformin exerts vascular anti-inflammatory effects *via* blocking the PI3K-Akt pathway and inhibiting NF-κB activation and nuclear translocation (54). An inflammatory vascular model was prepared by implanting polyester-polyurethane sponge into mice and which were then treated with metformin for 6 days. Metformin attenuated the main components of mouse fibrovascular tissue by regulating major components of inflammatory angiogenesis (macrophage recruitment, Hb content, transforming growth factor (TGF-β1) and collagen deposition) (61). In the rabbit AS model, metformin treatment decreased mRNA expression of adhesion molecules and inflammatory cytokines in the aorta. This result suggests that metformin may impede the development of AS by inhibiting macrophage infiltration and inflammatory responses (62). In C57BL/6 mice, methionine significantly up-regulated the levels of homocysteine (Hcy), TNF-α, H_2_S and IL-1β, and down-regulated the level of cystathionine γ-lyase (CSE). In THP-1 and raw264.7 cells, Hcy up-regulated the expression of DNA methyltransferase and increased the CSE promoter methylation. Whether in mice or in macrophages, metformin treatment can reduce the deleterious effects of methionine, which provides new insights into the actions of metformin to inhibit AS (63).

### NLRP3 Inflammasome

NLRP3 inflammasome (an innate immune signal complex) is mainly expressed as an inflammasome component in macrophages and is closely related to many diseases, including T2DM, AS, rheumatoid arthritis, gout and neurological diseases (64, 65). Various signals, including microbial molecules and abnormal accumulation of cholesterol crystals, can trigger NLRP3 assembly and activation, thereby promoting the conversion and secretion of proIL-1β and proIL-18 into mature forms (66). AS is a disease based on inflammation/lipid abnormalities (46). OxLDL and cholesterol crystals can activate NLRP3 inflammasome, and thereby the NLRP3 inflammasome may be a key intermediate link in the induction of inflammation by lipid metabolism disorders (67). Some studies in mouse hyperlipidemia models have shown that the NLRP3 inflammasome can cause AS, but it may require a second insult, such as oxidized mitochondrial DNA accumulation or impaired cholesterol efflux, which may cause serious systemic inflammatory (68). In macrophages or neutrophils, the activation of inflammasomes leads to the lysis of Gasdermin-D, which causes membrane pore formation, releases IL-18 and IL-1β, and ultimately leads to the formation of extracellular traps in neutrophils (NETosis) (68).

Compared with healthy controls, a significant up-regulation in NLRP3 mRNA and protein levels was found in monocyte-derived macrophages (MDM) of newly diagnosed T2DM subjects. Treatment with metformin for 2 months inhibited IL-1β maturation in MDM in patients with T2DM by AMPK activation. These data indicate that anti-diabetic treatment with metformin helps reduce inflammasome activation in T2DM (69).

Metformin can inhibit the NLRP3 inflammasome activation in apolipoprotein E (apoE) ^-/-^ mice and inhibit diabetes-accelerated AS, at least in part by activating AMPK and regulating thioredoxin-1/thioredoxin interacting protein (70). Furthermore, metformin can inhibit the expression and activation of NLRP3 in oxLDL-induced macrophages *via* AMPK and protein phosphatase 2A (PP2A) (71). Adenosine triphosphate (ATP) treatment results in AMPK activation, and host cells release ATP during bacterial infection as a stimulant of inflammasome activation. In LPS-stimulated mouse macrophages, ATP-stimulated inflammasome activation and pyrophosphorylation were inhibited by small interfering RNA-mediated AMPKα knockdown or compound C treatment. Moreover, the mortality of bacterial sepsis mice was increased by metformin administration, which may be because metformin promoted the activation of systemic inflammasomes in mice, as shown by increased serum and liver IL-1β levels (72).

The above studies indicate that metformin has the potential to inhibit NLRP3 inflammasome activity in chronic diseases (such as T2DM), while it may promote the activity of inflammasomes in acute diseases (such as bacterial infections).

### Oxidative Stress

Oxidative stress imbalance in tissues is closely related to the progression of many diseases, including AS, stroke, chronic wounds and cancer (73, 74). Oxidative stress and inflammation are two interrelated processes, forming a strong feedforward cycle, which promotes the development of atherosclerotic plaque (75, 76). During AS, mitochondrial oxidative metabolism, NADPH-oxidase, peroxidase, NO-synthase, cyclooxygenase and lipoxygenase produce macrophage ROS (75, 76). The antioxidant response of macrophages is the key to reducing ROS levels and protecting nucleic acids, proteins and mitochondria from oxidative damage (75, 76). In plaque macrophages, the mitochondrial transport of antioxidant glutathione (GSH) and the transcription of antioxidant genes are inhibited, which amplifies inflammation in the arterial wall (75, 76). It has been well established that NADPH-oxidase derived ROS from macrophages enhances the oxidation of LDL in the arterial wall, thereby promoting the formation of macrophage foam cells (75, 76). Antioxidant treatment is considered a promising therapy for the prevention of AS, but currently available antioxidants show quite limited clinical utility (8). This may be due to the various ways of inducing and inhibiting oxidation in the body and the single role of antioxidants, which did not achieve the expected effect (8, 77). Broad-spectrum ROS scavenging nanoparticles have a significant anti-atherosclerotic effect by decreasing local and systemic oxidative stress and inflammation (77). This suggests that pleiotropic antioxidants are more promising and will need to be further investigated.

Macrophages with Phosphatase and tensin homolog (PTEN) deficiency produce a sustained inflammatory microenvironment in which inducible nitric oxide synthase (iNOS)/nitric oxide (NO) as well as cyclooxygenase-2 (COX-2)/Prostaglandin E_2_ (PGE_2_) are produced in large amounts. Metformin can reduce inflammatory mediators in PTEN-deficient cells by inhibiting ROS production and Akt activation (78). Metformin reduces macrophage oxidative stress and inflammatory cytokine production by AMPK activation, but even after treatment with compound C, the residual activity of metformin is still significant (79). In addition, knockout of electron transport or calcium release activated channel (CRAC) in mouse alveolar macrophages can prevent particulate matter-induced inflammation and arterial thrombosis. Treatment of mouse or human alveolar macrophages with metformin can prevent the generation of complex III mitochondrial ROS induced by particulates, thereby inhibiting CRAC activation and IL-6 release (80). These studies have shown that metformin can inhibit oxidative stress in macrophages.

However, one study suggests that metformin may enhance oxidative stress. In the J774A.1 macrophage cell line, metformin reduced cholesterol biosynthesis from acetate, but at the same time significantly increased cellular oxidative stress, such as increased ROS production and decreased GSH level. Moreover, metformin inhibited cholesterol biosynthesis of macrophages which is at least partially related to metformin-induced oxidative stress (81). Chowdhury, AR et al. conjugated metformin with cationic triphenyl phosphate (TPP) to form Mito-Metformin to selectively target mitochondria. In HCT116 adenocarcinoma cells, C2C12 skeletal muscle cells and Raw264.7 macrophages, Mito-Metformin induced the generation of ROS in mitochondria by acting on complex I (82). These studies indicate that metformin may have the effect of activating oxidative stress in macrophages, but this may not affect the protection of metformin on the function of macrophages.

Mitochondrial damage plays an important role in the pathogenesis of obesity, diabetes, and CVD (83, 84). Mitochondrial function declines with age. The destruction of mitochondrial function is the result of a variety of intracellular and extracellular stresses (84). Many studies have shown that metformin improves mitochondrial function and inhibits oxidative stress (83, 85). Other studies reported that metformin inhibits mitochondrial function and enhances oxidative stress. This however, may be attributed to metformin overdosing. For example, in a study where metformin reduced cholesterol biosynthesis while enhancing oxidative stress (81), the drug dosage was 2-5 mM, which is significantly higher than the typical.

### Foam Cell Formation

Foam cells rich in cholesteryl esters are a sign of atherosclerotic plaque (86). Plasma-derived lipoproteins are modified in the inner membrane and are absorbed by macrophages to form lipid-filled foam cells, causing the formation of atherosclerotic lesions. Foam cells lack endocytosis and have insufficient inflammatory ablation ability, which maintains the progression of the disease. This results in the accumulation of secondary necrotic macrophages and foam cells, and the formation of late lesions with necrotic lipid cores, causing plaques to be vulnerable to rupture (87).

In macrophages, metformin inhibits the accumulation of cholesterol induced by 3-deoxyglucosone (3-DG) (88), oxLDL (89), acetate (81), LPS (90) and palmitic acid (91).

HDL-mediated cholesterol efflux is a rate-limiting step in reverse cholesterol transport (RCT). In macrophages, glycated HDL particles (glycosylated by 3-DG) cannot be effective as receptors for ATP-binding cassette transporter G1 (ABCG1)-mediated cholesterol efflux. Metformin can restore cholesterol efflux mediated by glycated HDL (88). Another similar study indicated that metformin reduced oxLDL-stimulated cholesterol accumulation and the formation of foam cells *via* promoting cholesterol efflux to HDL, which may be related to up-regulation of ABCG1. In addition, metformin increases IL-10 secretion inhibited by oxLDL, which is an important anti-foam cell forming factor in AS (89).

In the J774A.1 macrophage cell line, metformin reduced cholesterol biosynthesis rate from acetate (81). In addition, metformin inhibits LPS-induced THP-1-derived foam cell formation and reduces adipogenic differentiation-associated protein (ADRP) expression (90). Metformin also reduces palmitate-induced lipid accumulation in macrophages *via* reducing the transcription of fatty acid binding protein 4 (FABP4) mediated by forkhead box transcription factor O1 (FOXO1) (91).

Combined with other drugs, metformin may have enhanced efficacy and reduced adverse reactions. Liver X receptor (LXR) agonist T317 can improve AS, but at the same time induces fatty liver. In a high fat diet (HFD) fed apoE^-/-^ mice, co-administration of metformin and T317 inhibits the development of AS, including down-regulation of monocyte adhesion and macrophages cell proliferation, and up-regulation of ABCA1/ABCG1 expression. Metformin blocks T317-induced fatty liver *via* reducing T317-stimulated hepatic LXRα nuclear translocation, adipogenic gene expression and activating AMPKα. This suggests that treatment with metformin and T317 may be a new strategy to decrease foam cell formation and AS (92).

Although there is a positive effect on the reverse transport of cholesterol in macrophages, metformin may have no significant effect on the reverse transport of intestinal cholesterol. The effect of dipeptidyl peptidase-4 inhibitor (DPP-4i) sitagliptin and metformin on RCT was studied using obese insulin-resistant CETP-apoB100 transgenic mice. Sitagliptin (rather than metformin) increased fecal cholesterol excretion by 132%, indicating that sitagliptin promotes RCT by reducing intestinal cholesterol absorption (93).

Although there is no significant effect on the reverse transport of intestinal cholesterol, the above studies indicate that metformin may decrease lipid accumulation in macrophages and exert a positive effect in AS in patients with the MetS.

### M1/M2 Macrophage Polarization

In addition to the first line of defense, macrophages also exert a very important effect in maintaining the homeostasis of various tissues and organs (94, 95). In response to extrinsic factors from a given tissue, macrophages activate different functional programs to generate polarized macrophage populations, which are responsible for inducing inflammation against microorganisms, removing cell debris, and tissue repair (96). The role of macrophages in AS is thought to be inseparable from the polarization and phenotypic expression of macrophages. In addition, the role of macrophages in AS depends not only on the function of different macrophage phenotypes, but also on the relative proportion of different phenotypes in atherosclerotic plaques. Studies on AS therapy have shown that the decrease in plaque size and the increase in stability are partly attributable to the regulation of macrophage polarization (97). M2 macrophages are associated with regression of AS. The M2 macrophages can produce the IL-10 and TGF-β, thereby eliminating dying cells and debris through endocytosis, and promoting tissue remodeling and repair through collagen formation (98). Platelets represent an important cell type that mediates inflammation and immune processes in AS, mainly by secreting chemokines when platelets are activated. For example, CXCL4 binds to CCL5 to induce monocyte adhesion, thereby promoting the transdermal effect of monocytes into the subendothelial space. CXCL4 also induces monocyte differentiation and forms a specific macrophage phenotype (M4) (99). In summary, changing macrophage proportions and adjusting macrophage polarization in the plaque represents a new treatment frontier for the therapy of AS.

In a study involving 30 normal-weight healthy adult volunteers, 30 obese volunteers, 20 obese newly diagnosed patients with diabetes, as well as 30 metformin-treated obese patients with diabetes peripheral blood mononuclear cells (PBMCs) were isolated and polarization markers were measured. The results showed CD68 marker was increased in obesity and in T2DM. The levels of CD11b, CD11c, CD163 and CD169 in T2DM patients were reduced, and CD11c in obese volunteers was significantly inhibited. In addition, the increased expression of TNFα, iNOS, IL-6, CD16, CD36, and CD206 suggests that the M1-like phenotype of macrophages is observed in the T2DM. With metformin treatment, TNFα, iNOS, IL-6, CD11c, CD36, CD169 and CD206 levels in T2DM patients were restored to levels of lean volunteers. The results of this study suggest that PBMCs in T2DM express a different pattern of phenotypic markers (representing metabolically activated macrophage (MMe)-like cells), which is not the pattern normally found in M1 or M2-like macrophages, and further that metformin can reduce circulating MMe-like cells (100).

In HFD-fed C57/6J male mice, metformin administration for 7 weeks not only decreased blood levels of TNF-α and IL-6, but also decreased M1 macrophage marker (MCP1 and CD11c) in AT. This study demonstrates that metformin regulates macrophages phenotype to M2 by activating AMPK, which decreases low-grade inflammation in obesity (101). The Src homology 2 (SH2) domain-containing protein tyrosine phosphatase 1 (SHP-1) is a negative mediator of inflammation. In an obese mouse model, metformin effectively polarizes AT macrophages to an anti-inflammatory state *via* indirectly inhibiting SHP-1 expression, thereby downregulating NF-κB, STAT1, CD80, CD86, TLR2, TLR4 and inhibiting inflammation of AT (102). Acute AMPK activation has exacerbated ischemic brain injury, but the clinical application of metformin reduced the incidence of stroke. Chronic metformin administration after stroke improves functional recovery after cerebral artery occlusion (MCAO) in mice by AMPK-dependent microglia/macrophage M2 polarization. Regulation of microglia/macrophage polarization may represent a promising treatment for stroke (103). In apoE^-/-^ mice, pharmacological AMPK activation (such as metformin) inhibits the formation of atherosclerotic plaque by inducing macrophage M2 polarization, reducing plasma lipids peroxidation and inflammatory cytokines expression (104).

In a cellular model, metformin induces RAW264.7 macrophages with/without LPS stimulation to the M2 phenotype, and Notch1 signaling may be involved in the regulation process of metformin on the polarization of macrophages (105). Metformin affects the phenotype of macrophages and ameliorates the activity of glutathione peroxidase, superoxide dismutase and catalase (79). However, studies have also shown that metformin specifically attenuates the production of pro-inflammatory cytokines without reducing M1/M2 differentiation (55).

### Monocyte Differentiation Into Macrophage

**Figure 2:**
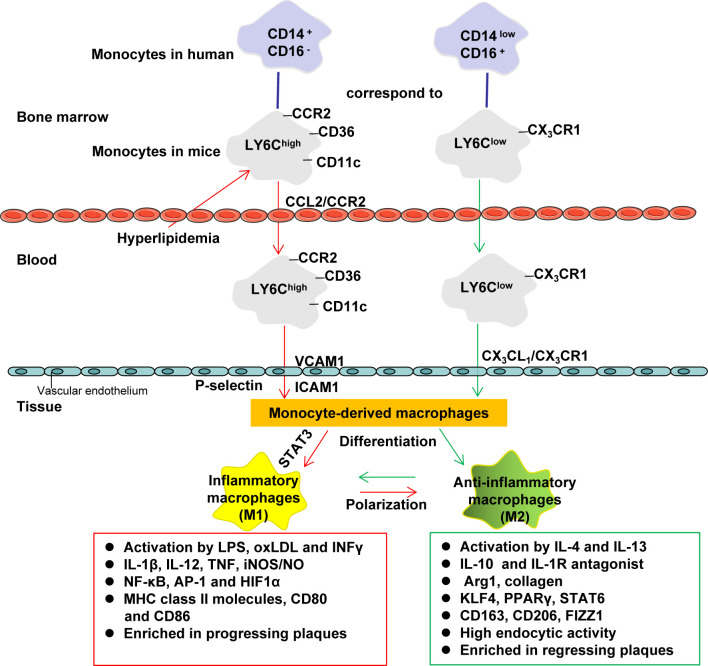
Monocyte differentiation and macrophage polarization. Monocytes are derived from the hematopoietic precursors of bone marrow or spleen and produce the classic lymphocyte antigen 6C (LY6C) ^high^ in mice (corresponding to human CD14^high^ CD16^low^ monocytes), and nonclassical-LY6C^low^ in mice (corresponding to human CD14^low^ CD16^high^ monocytes). LY6C^high^ monocytes highly express CCR2, which can be recruited by chemokines (such as CCL2) to inflammatory sites (including atherosclerotic plaques) to play a pro-inflammatory effect (it is considered to be the precursor of M1 macrophages). Hypercholesterolemia promotes the production of LY6C^high^ monocytes by inducing the proliferation of bone marrow precursors. LY6C^low^ monocytes (considered to be the precursor of M2 macrophages) highly express CX_3_CR1. These monocyte subpopulations use different chemokine-chemokine receptor pairs to penetrate into the inner membrane, and then differentiate into macrophages in the inner membrane (LY6C^high^ is more likely to differentiate into M1 macrophages, while LY6C^low^ is more likely to differentiate M2 macrophages). M1 macrophages amplify the inflammatory effect by secreting pro-inflammatory cytokines. M2 macrophages help tissue repair by secreting anti-inflammatory cytokines and collagen. AP-1, activator protein 1; Arg1, arginase 1; CCL, C−C motif chemokine; CCR, C-C chemokine receptor; CD, cluster of differentiation; FIZZ1, found in inflammatory zone 1; HIF1α, hypoxia-inducible factor 1α; ICAM1, intercellular adhesion molecule-1; INFγ, interferon-γ; iNOS, inducible nitric oxide synthase; IL, Interleukin; IL-1R, Interleukin 1 receptor; KLF4, Krüppel-like factor 4; LPS, lipopolysaccharide; NO, nitric oxide; NF-κB, nuclear factor kappa B; PPAR, peroxisome proliferator activated receptor; STAT, Signal transducer and activator of transcription; VCAM1, vascular cell adhesion protein 1.

Monocytes are derived from the hematopoietic precursors of bone marrow or spleen and produce the classic lymphocyte antigen 6C (LY6C) ^high^ in mice (corresponding to human CD14^high^ CD16^low^ monocytes), and nonclassical-LY6C^low^ in mice (corresponding to human CD14^low^ CD16^high^ monocytes) (49, 106). LY6C^high^ monocytes highly express CC chemokine receptor 2 (CCR2), and is considered to be the precursor of M1 macrophages. LY6C^high^ monocytes can be recruited by chemokines to atherosclerotic plaques and play a pro-inflammatory effect (49). Hypercholesterolemia promotes the production of LY6C^high^ monocytes by inducing the proliferation of bone marrow precursors (107). LY6C^low^ monocytes (considered to be the precursor of M2 macrophages) highly express chemokine receptor CX3CR1 (49). These monocyte subpopulations use different chemokine-chemokine receptor pairs to penetrate into the inner membrane, and then differentiate into macrophages in the inner membrane (106). The differentiation of LY6C^high^ monocytes to M1 macrophages promotes the inflammatory environment in the blood vessel wall to exacerbate AS (108) (**Figure 2**).

Forty-four T2DM patients with acute myocardial infarction were divided into two groups according to the hypoglycemic drugs taken, including 21 cases of metformin and 23 cases of short-acting insulin. Metformin treatment resulted in a faster reduction in sCD40L compared to insulin treatment, which helped to improve the prognosis in this cohort. Mechanistic studies suggest that the inhibitory effect of metformin on sCD40L levels may be due to reduction of Akt phosphorylation, which is responsible for activating immune response genes that cause platelet activation and monocyte differentiation into macrophages cell capable of producing AS (109).

In the AS model, metformin inhibits monocyte differentiation. In a diet-induced rabbit AS model, administration of metformin inhibited rabbit monocytes from differentiating into macrophages and inflammatory responses and decreased aortic mRNA expression of adhesion molecules and inflammatory cytokines (62). In ApoE^-/-^ mice, the AMPK-STAT3 axis exerts a key effect in modulating the differentiation of monocytes-macrophages. Metformin reduces STAT3 phosphorylation by increasing AMPK activity and inhibits monocyte differentiation into macrophages (40).

### Apoptosis

During the formation and development of atherosclerotic lesions, lipid-filled macrophages form foam cells, accumulate and eventually undergo apoptotic death. Further aggregation of apoptotic foam cells may lead to secondary necrosis as well as the formation of a necrotic lipid core, which makes the plaque unstable and prone to rupture (**Figure 1**). Thus, non-lipid-filled macrophages, as the main phagocytic cells in atherosclerotic lesions, need to effectively remove apoptotic foam cells (110).

Apoptosis of OxLDL-stimulated macrophages contributes to the development of AS. Metformin reduces oxLDL-stimulated macrophage lipid uptake and prevents macrophage apoptosis. Possible mechanisms involve inhibition of ER stress (reduction of eukaryotic translation initiation factor 2A, C/EBP homologous protein and glucose regulatory protein expression), reversal of mitochondrial membrane potential loss and cytochrome c (cyto-c) release, as well as regulation of scavenger receptor expression (111). In addition, inflammatory aging is related to the progression of diabetes complications. Senescence-related secretory phenotype (SASP) is the main factor resulting in inflammatory senescence, and macrophages are important SASP-carrying cells. In macrophages, high glucose can induce cell senescence and secretion of SASP factors through the phosphorylation of domain (CARD)-containing 4 (NLRC4), and further stimulate the NF-κB/Caspase-1 cascade through the interferon regulatory factor 8 (IRF8) pathway. Knockout of NLRC4 or IRF8 inhibited cell senescence and SASP caused by hyperglycemia in macrophages. Under high glucose conditions, metformin treatment can inhibit NLRC4 phosphorylation and significantly reduce cell senescence and SASP (112).

Significant progress has been made in the study of metformin and macrophage function, but there are still some limitations to these investigations. First of all, most of the studies on metformin and AS looking at improving the function of macrophages are pre-clinical studies (**Table 2**). Compound C (an AMPK inhibitor) and 5-amino-1-β-D-furanofuranosyl-imidazole-4-carboxamide (AICAR, as AMPK activator) are often used in mechanistic pharmacological research; however, compound C can produce off-target effects by inhibiting other protein kinases (124–126). As an AMPK agonist, AICAR may not be a sufficiently specific enough tool to address the index questions (127). The M1/M2 polarization effect of AICAR on macrophages has no difference in $AMPK\alpha_{2}^{+/+}$ and $AMPK\alpha_{2}^{-/-}$, which indicates that the M2 polarization effect induced by AICAR is at least partially independent of AMPK (128). Therefore, it is necessary to use more specific AMPK activators and inhibitors to study the role of metformin. Importantly, the maximum dose of oral metformin in clinical practice is 2.5–3 g per day, which is approximately 35–42 mg/kg (129, 130). In humans, the plasma concentration range of metformin after passing through the liver is usually 10–40 μM (130). When we collated the studies of metformin and macrophage function in atherosclerotic diseases, we found that the dose of metformin of some studies was much higher than the highest clinical dose (**Table 2**) (72). Such studies have a role in exploring the mechanism of action of metformin, but such studies may lack practical clinical significance.

**Table 2:** Clinical, animal and cellular studies of metformin on macrophages associated with atherosclerotic diseases.

Functions	Models or subjects	Mechanisms	Results	Conclusions
*Inflammation*	DM patients +metformin(n=498) or + Sulfonylurea(n=172), non-diabetic patients with congestive heart failure+metformin (n=20) or + Placebo (n=13)	↓neutrophils to lymphocytes (NLR), ↓CCL11	↓inflammation in diabetic and non-diabetic patients	The anti-inflammatory effect of metformin has nothing to do with diabetes status. The study may accelerate the study of the effect of metformin in non-diabetic CVD (55).
	PCOS (n = 83), Controls (n = 39), and PCOS+ metformin (n=21)	↓ASAA	↓intima-media	Metformin treatment can reduce serum ASAA in women with PCOS (57).
	ARDS mice Metformin (50 mg/kg) Alveolar macrophages (NR8383) cells, metformin (40 μg/mL)	↓miR-138-5p ↑ SIRT1 ↓p-p38, p-ERK and p-NF-κB	↓LPS-induced deaths ↓IL-6, IL-1β, IL-17 and TNF-α	Metformin can reduce the LPS-induced lung damage *via* decreasing miR-138-5p expression, increasing the expression of its target gene SIRT1 and inhibiting MAPK pathway (58).
	LPS treated macrophages, metformin (10 mM)	↑AMPK, ↓NF-κB	↓CXCL10 and CXCL11	Metformin inhibits LPS-stimulated chemokines expression *via* AMPK and NF-κB signaling pathways (53).
	ECs, macrophages and SMCs, Metformin (20 μM)	↓PI3K-Akt ↓NF-κB	↓IL-6 and IL-8	Metformin can block NF-κB by inhibiting the PI3K-Akt pathway, thereby exerting a direct vascular anti-inflammatory effect (54).
	HFD fed rabbit, Metformin (200 mg/kg/day) monocytes, macrophages metformin (200 μg/mL)	↓inflammatory cytokines and adhesion molecules	↓AS ↓the adhesion of monocytes, inflammatory response of macrophages	Metformin may inhibit the development of AS *via* inhibiting macrophage infiltration and inflammation (62).
*Inflammation*	LPS-stimulated endotoxemia mice, metformin (250 or 500 mg/kg, twice daily), ob/ob mice (250 mg/kg, twice daily) LPS treated macrophages, metformin (0.5, 1, 2, 4 mM)	↑AMPK and ATF3 NF-κB enrichment on TNF-α and IL-6 promoters caused by LPS were replaced by ATF3	↓TNF-α and IL-6	AMPK activation and induction of ATF3 are potential mechanisms for metformin to exhibit anti-inflammatory effect in macrophages (113).
	LPS induced macrophages, metformin (10 mM)	↑AMPK ↓NF-κB	↓CXCL10 and CXCL11, ↓IL-1 and IL-6	Metformin reduces LPS-stimulated chemokine expression *via* AMPK and NF-κB signaling pathways (53).
	AGEs induced mouse BMDM Metformin (2μM)	↑AMPK ↓NF-κB	↓CD86) (M1 marker), ↑CD206 (M2 marker) and IL-10	Metformin partially reduces AGEs- stimulated the inflammatory response of mouse macrophages *via* AMPK activation and RAGE/NFκB pathway inhibition (114).
	HFD fed mice, metformin (150 mg/kg/d) LPS-induced BMDM, metformin (500 µM)	↑AMPK ↓JNK1 and NF-κB	↓liver steatosis ↓fat deposition ↓pro-inflammatory cytokines and lipogenic enzymes	Metformin mainly prevents obesity-related NAFLD by directly reducing liver cell fat deposition and inhibiting the inflammatory response of liver cells and macrophages (115).
	Acetaminophen treated mice, metformin (350 mg/kg) HMGB1 treated macrophages, metformin (0.1~10 mM)	Binds directly to the C-terminal acid tail of HMGB1.	↓inflammatory response	Metformin inhibits inflammation *via* reducing the extracellular activity of HMGB1 (116).
*Inflammation*	Neutrophils from patients with ARDS, metformin (500 µM/L)	↑ AMPK Neutralization of HMGB1 in BAL fluid	Neutralization of HMGB1 in BAL fluid or activation of AMPK in macrophages in BAL fluid improved cell swelling and NETs clearance.	Neutralizing HMGB1 or restoring AMPK activity with metformin represents a promising therapeutic strategy to reduce persistent lung inflammation of ARDS (117).
	LPS-treated mice, metformin (50, 100 mg/kg), LPS-treated macrophages, metformin (1, 5, 10 mM)	↑ AMPK ↓HMGB1.	↓serum levels of HMGB1, IL-1β, TNF-α and myeloperoxidase activity in the lung. ↑the survival rate	Metformin improves the survival rate of the lethal endotoxemia mouse model *via* inhibiting the release of HMGB1. AMPK activation is one of the mechanisms causing HMGB1 secretion inhibition (118).
	Methionine treated mice, Hcy treated macrophages, metformin (12.5, 25 and 50 μM)	↑CSE expression	↓levels of Hcy, TNF-α and IL-1β,	Metformin treatment reduces the harmful effects of methionine (63).
*Inflammasome*	ATP treated macrophages, metformin (2 mM) bacterial sepsis mice, metformin (250 mg/kg)	↑AMPK ↑inflammasomes	↑mortality of mice with bacterial sepsis. ↑the activation of systemic inflammasomes (such as increased IL-1β level in blood and liver).	These results indicate that AMPK signal transduction positively regulates ATP-cuased inflammasome activation and pyrophosphorylation in macrophages (72).
	oxLDL treated macrophages, metformin (80μM)	↑AMPK and PP2A, ↓NF-κB	↓NLRP3 inflammasome	Metformin inhibited expression and activation of NLRP3 in oxLDL-induced macrophages *via* AMPK and PP2A (71).
*Inflammasome*	Diabetic mice, Metformin (300 mg/kg/d)	↓disorder of thioredoxin-1/thioredoxin interacting protein. ↓ROS and NLRP3 inflammasome	↓metabolic disorders and AS	Metformin can inhibit the NLRP3 inflammasome activation in ApoE^-/-^ mice and inhibit diabetes-accelerated AS, at least in part by activating AMPK and regulating thioredoxin-1/thioredoxin interacting protein (70).
	Control (n=57), T2DM patients (n=47), Among 47 diabetic patients, 11 patients received metformin(500–1,000 mg/day).	↑AMPK	↓the maturation of IL-1β in MDM of T2DM patients	NLRP3 inflammasome is activated in MDM of T2DM patients, and metformin administration helps to regulate the activation of inflammasomes in T2DM (69).
*Oxidative stress*	Macrophages (shPTEN cells), metformin (5 to 40 mM)	↓Akt and ROS	↓iNOS/NO and COX-2/PGE_2, _ ↑apoptosis	In shPTEN cells, Metformin can reduce the diffusion of inflammatory mediators and cell growth by inhibiting Akt activation and ROS production (78).
	LPS treated Peripheral blood monocytes, metformin (0.02 and 2 mM)	↑AMPK ↑ superoxide dismutase, glutathione peroxidase, catalase ↓malondialdehyde	↓ROS and inflammatory cytokines (such as iNOS)	Metformin can improve diabetes by lowering blood glucose, reducing the oxidative stress, inflammatory cytokines, and inducing phenotypic changes of macrophages (79).
	macrophages, metformin (2-5 mM)	↓Glutathione ↑paraoxonase 2 ↑ cellular ROS	↓cholesterol content and biosynthesis rate Antioxidants decreased metformin-induced ROS and cancelled the inhibitory effect of cholesterol biosynthesis.	The inhibitory effect of metformin on cholesterol biosynthesis is at least partially related to metformin-induced ROS in macrophages (81).
	macrophages, C2C12 skeletal muscle cells and HCT116 adenocarcinoma cells, Mito-Metformin	↑calcineurin and Cn-dependent retrograde signaling pathway	↑ ROS in mitochondria	The retrograde signal induced by Mito-Metformin is through the activation of the Ca2/Cn pathway (82).
*Foam cell formation*	ApoE^-/-^ mice fed with HFD, metformin(260 mg/kg)	↑AMPK, ↑ABCA1 and ABCG1 in macrophages, ↑LCAT and SR-B1 in liver. ↑ M2 polarization ↑paraoxonase 1 ↓myeloperoxidase	↑RCT ↓blood lipids peroxidation, ↓inflammatory cytokines expression ↓atherosclerotic plaque.	AMPK activators promote the anti-atherosclerotic properties of HDL and attenuate AS (104).
	3-DG incubated macrophages, metformin (100 mM)	↑the glycated HDL-mediated cholesterol efflux Exogenous HDL reduces the expression of ABCG1 mRNA and protein, but glycosylation makes HDL lose this effect.		Glycated HDL particles cannot effectively act as ABCG1-mediated cholesterol efflux receptors. Metformin may be a drug candidate to improve cholesterol efflux (23).
	LPS and oxLDL induced macrophages Metformin (100 or 200 μM/L)	↓ ADRP	↓LDs in the foam cells	Metformin can reduce the formation of THP-1 derived foam cells caused by LPS, decrease the ADRP expression and intracellular lipid accumulation (90).
	palmitic acid (PA) treated macrophages, Metformin (100, 250, 500μM)	↓FOXO1 ↓FABP4 ↑ CPT-1.	↓lipids accumulation in macrophages	Metformin reduces the lipids accumulation in macrophages *via* reducing FOXO1-mediated FABP4 transcription (91).
	oxLDL treated macrophages, metformin (15 μM)	↑ABCG1 ↑ outflow of cholesterol to HDL ↑IL-10 secretion	↓cholesterol accumulation and the formation of foam cell	The study highlights the therapeutic potential of metformin to target macrophage cholesterol efflux, which may reduce foam cell formation (89).
*Foam cell formation*	ApoE^-/-^ mice, Co-treatment with T317 and metformin (100 mg/day/kg)	↓macrophages/foam cells in the arterial wall ↑ABCA1/ABCG1. Metformin activates AMPKα and reduces T317-stimulated hepatic LXRα activation and lipogenic gene expression.	Co-administration increases the stability of the lesion block T317-caused fatty liver	Co-administration of metformin and T317 can improve AS without activating adipogenesis, which indicates that this combination may be a new method to inhibit AS (92).
	mouse BMDM and primary human MDMs, metformin (10 μM)	↑AMPK ↑ATF1.	Induced heme oxygenase and LXR jointly induce the Mhem phenotype.	Heme (10 μM) activates AMPK, and the downstream ATF1-induced heme oxygenase and LXR jointly induce the Mhem phenotype. (119).
	LPS induced macrophages, metformin (1-3 mM)	↓NF-κB	apoE expression↑	Metformin is a potential adjuvant in the treatment strategy of AS (120).
	HFD fed mice, metformin (250mg/kg) primary hepatocytes, metformin(0.5 mmol/L)	↓multiple binding sites of phase 2 (transcription repressor) occupancy within ABCG5/8 site	↑expression of ABCG5/8 and BSEP ↑initial clearance of 3H-cholesteryl ester HDL from the blood.	Metformin may provide some support for cardiovascular benefits by increasing RCT, and AMPK activation inhibition may mediate anti-atherosclerotic effects by increasing ABCG5/8 expression (121).
*M1/M2 polarization*	PBMCs were isolate from 30 normal-weight healthy adult volunteers, 30 obese volunteers, 20 obese newly diagnosed diabetic patients, as well as 30 metformin-treated obese diabetic patients.	↑CD68 marker in obesity and in T2DM. ↓CD11b, CD11c, CD163 and CD169 in T2DM patients ↑TNFα, iNOS, IL-6, CD16, CD36, and CD206 in the T2DM Metformin restored TNFα, iNOS, IL-6, CD11c, CD36, CD169 and CD206 in T2DM patients to levels equivalent to those of lean volunteers.		PBMCs in T2DM patients express a different pattern of phenotypic markers (represent metabolically activated macrophage (MMe)-like cells), which is not the pattern normally found in M1 or M2-like macrophages, and metformin can reduce circulating MMe-like cells (42).
	Olanzapine reated rats, metformin (300 mg/kg/day)	↓body weight and IR, ↓macrophage polarization and pro-inflammatory factors.		Metformin may reduce the IR caused by olanzapine by inhibiting the polarization and inflammation of macrophages in white AT (43).
*M1/M2 polarization*	HFD fed mice, metformin (300 mg/kg/d) In palmitate stimulated BMDM, metformin (2 mM)	↑ AMPK, ↓IL-6 and TNF-α ↓CD11c and MCP1 (M1 markers) in AT ↓proportion of M1 macrophages, ↑the proportion of M2 macrophages		Metformin can regulate the polarization of macrophages to anti-inflammatory M2 and improve low-grade inflammation in obesity by activating AMPK (23).
	Obese mice, metformin (300 mg/kg)	↓SHP-1 and ↑insulin sensitivity. ↑anti-inflammatory macrophages AT	↓CD80, CD86, TLR2, TLR4, NF-κB, STAT1 and other inflammatory markers ↓inflammation of AT	Metformin exerts its insulin sensitization effect by inhibiting the activity and expression of SHP-1 (102).
	Ldlr^-/-^ hyperlipidemia mice, metformin (in drinking water, 1mg/mL) Mouse BMDM, metformin (10 μM)	↑AMPK/ATF1 ↑M2 marker genes, ↓ iNOS	↓atherosclerotic lesions. ↑LXRβ, Hmox1, ApoE, ABCA1, PDGF and IGF1	metformin can activate the AMPK-ATF1-M2-like pathway in macrophages. These findings support the clinical trials of metformin in non-diabetic patients with high risk of AS (122).
	MCAO mice, metformin (50 mg/kg/day)	↑ AMPK, microglia/macrophages tend to M2 phenotype	↑ functional recovery, ↑neurogenesis and angiogenesis	Chronic metformin treatment after stroke improves functional recovery through AMPK-induced M2 polarization (103).
	macrophages with/without LPS, metformin(1, 5, 10 mM)	↑IL-4, IL-10, arginase 1 (Arg1) and lectin-1 (Mgl1) ↓Notch1, TNF-α, IL-1β and IL-6.		Metformin regulates the M2 phenotype of RAW264.7 macrophages with/without LPS. The Notch1 signal may play a vital role (23).
*Monocyte differentiation*	Forty-four subjects with AMI and T2DM (metformin, n=21; short-acting insulin, n=23)	↓Akt ,	↓sCD40L level	Metformin therapy in patients with AMI and T2DM can cause a faster decline in sCD40L, which may help improve the prognosis of this cohort (109).
*Monocyte differentiation*	HFD-fed ApoE^-/-^ mice, metformin (260 mg/kg)	↓CCR2 expression	↓number of Ly6Chi monocytes in circulation as well as atherosclerotic plaques.	AMPK activation decreases the development of AS induced macrophages accumulation in ApoE^-/-^ mice *via* reudcing the CCR2 expression, thereby preventing CCR2-induced migration of Ly6C^high^ monocytes (123).
	Ang-II induced ApoE^-/-^ mice, Metformin (100 mg/kg/day)	↑AMPK, ↓ STAT3	↓the infiltration of monocytes, ↓atherosclerotic plaques and aortic aneurysms	AMPK activators reduce the differentiation of monocytes into macrophages by regulating AMPK-STAT3 axis (40).
*Apoptosis*	oxLDL treated metformin, macrophages (0.1, 0.3, 0.5, 1 mM)	↓scavenger receptors, including CD36 and SRA ↓expression of ER stress marker proteins (such as EIF2A and CHOP) ↓oxLDL-induced Δψ_m_ loss and cyto-c release.	↓lipid uptake ↓the apoptosis of macrophages	Metformin can prevent oxLDL-caused macrophage apoptosis and inhibit lipid uptake of macrophage (111).

In this table, we describe that metformin plays a role in atherosclerosis by regulating monocyte/macrophage function, including cell function, objects, mechanisms, results and conclusions. ↑Represents increase or activation. ↓Represents to reducion or inhibition. The corresponding abbreviations are as follows: ABCA1, ATP-binding cassette transporter A1; ABCG1, ATP-binding cassette transporter G1;ADRP, adipose differentiation-related protein; AMPK, AMP-activated protein kinase; AGEs, advanced glycation end products; AMI, acute myocardial infarction; Ang-II, angiotensin II; ApoE, apolipoprotein E; ARDS, acute respiratory distress syndrome; ASAA, acute-phase serum amyloid A; AS, atherosclerosis; AT, adipose tissue; ATF3, transcription factor 3; BAL, bronchoalveolar lavage; BM, bone marrow; BMDM, bone marrow-derived macrophages; BSEP, bile salt export pump; CXCL10, C-X-C motif ligand; CCR2, CC chemokine receptor 2; CD, cluster of differentiation; CHOP, C/EBP homologous protein; CSE, cystathionine γ-lyase; COX-2, cyclooxygenase 2;CPT-1, carnitine palmitoyl transferase I; 3-DG, 3-deoxyglucosone; DM, diabetes mellitus; ECs, endothelial cells; EIF2A, eukaryotic translation initiation factor 2A; ERK, extracellular signal-regulated kinase; ER, endoplasmic reticulum; FABP4, fatty acid binding protein 4; FOXO1, forkhead box transcription factor O1; HFD, homocysteine (Hcy,; high-fat diet; HMGB1, high-mobility group box 1; Hmox1, Heme oxygenase 1; H_2_S, hydrogen sulfide; IGF1, insulin growth factor 1 IL, Interleukin; iNOS, inducible nitric oxide synthase; JNK1, c-Jun N-terminal kinase 1; LCAT, lecithin:cholesterol acyltransferase; Ldlr, low-density lipoprotein receptor; LPS, lipopolysaccharide; LXR, Liver X receptor; MAPK, mitogen activated protein kinase; MCAO, middle cerebral artery occlusion; MCP1, monocyte chemoattractant protein 1; MDMs, monocyte-derived macrophages; Δψ_m_, mitochondrial membrane potential; NAFLD, non-alcoholic fatty liver disease; NETs, neutrophil extracellular traps; NF-κB, nuclear factor-κB nucleotide-binding oligomerisation domain receptor, pyrin domain containing (NLRP)3, the ratio of neutrophils to lymphocytes (NLR), NO, nitric oxide; oxLDL, oxidized low-density lipoprotein; PA, palmitic acid; PBMC, peripheral blood mononuclear cell; PCOS, Polycystic ovary syndrome; PDGF, platelet-derived growth factor; PGE_2_, prostaglandin E_2_; PI3K, phosphatidylinositol 3-kinase; AKT, protein kinase B; PMA, phorbol 12-myristate 13-acetate; PTEN, phosphatase and tensin homolog; RAGE, receptor for advanced glycation end products; ROS, reactive oxygen species; SH2, Src homology 2; domain-containing protein tyrosine phosphatase 1 (SHP-1); SIRT1, Sirtuin-1; SMCs, smooth muscle cells; SRA, scavenger receptor class A; SR-B1, scavenger receptor class B type 1; STAT, signal transducer and activator of transcription; TNF-α, tumor necrosis factor-α; TIP47, tail-interacting protein; TLR, Toll-like receptor.

## Molecular Targets of Metformin

### AMPK

Adenosine monophosphate activated protein kinase (AMPK) plays a very critical role in the many beneficial effects of metformin, and in the protection from AS afforded by metformin. Studies have shown that long-term treatment with metformin to activate AMPK can reduce atherosclerotic calcification and inhibit Runt-related transcription factor (Runx2) expression in ApoE^-/-^ mice noting that Runx2 is an important promoting factor of vascular calcification in mice. On the other hand, metformin has little effect on atherosclerotic calcification in ApoE^-/-^/AMPKα1^-/-^ mice (39).

The differentiation of monocytes into macrophages is a key event that exacerbates AS *via* enhancing the inflammatory environment in the blood vessel wall (40). Hyperlipidemia usually reduces AMPK activity and increases CC chemokine receptor 2 (CCR2) expression. CCR2 controls the migration of Ly6C^high^ monocytes from the bone marrow (BM) to blood, which contributes in the accumulation of macrophages in the progression of AS. ApoE^-/-^ mice were fed with HFD and then received AMPK activator (metformin, A769662 or AICAR) treatment for 10 weeks. The AMPK activators decreased the Ly6C^high^ monocyte migration by reducing CCR2 protein expression. At the same time, AMPK activators decreased AS-induced macrophage accumulation in ApoE^-/-^ mice *via* reducing CCR2 expression (113). Similarly, in ApoE^-/-^ mice, metformin and AICAR can partially reduce monocyte infiltration, thereby reducing Ang-II stimulated atherosclerotic plaque formation and aortic aneurysms (40). During phorbol 12-myristate 13-acetate (PMA) mediated differentiation of monocytes into macrophages, AMPK activity decreases and pro-inflammatory cytokine levels increase. AMPK activators metformin and AICAR reduce monocyte differentiation stimulated by PMA and the accompanying pro-inflammatory cytokine expression. By reducing the phosphorylation of STAT3, metformin and AICAR inhibit the differentiation of monocytes to macrophages by increasing AMPK activation (including in the absence of PMA). These findings indicate that the AMPK-STAT3 axis exerts a key role in modulating the differentiation of monocytes into macrophages, and AMPK activators reduce STAT3 phosphorylation by increasing AMPK activity and inhibiting monocyte differentiation (40).

In murine macrophages, metformin inhibits LPS-stimulated IL-6 and TNF-α production, and simultaneously induces activation of transcription factor 3 (ATF3). ATF3 gene silencing reversed the inhibitory effect of metformin on LPS-stimulated pro-inflammatory cytokine production, and at the same time annulled the inhibitory effect of metformin on MAPK phosphorylation. After metformin treatment, the NF-κB enrichment on IL-6 and TNF-α promoters stimulated by LPS was replaced by ATF3. AMPK gene silencing attenuates all the beneficial effects of metformin (including ATF3 activation, pro-inflammatory cytokine suppression and MAPK inhibition). These findings show that anti-inflammatory effect of metformin in macrophages is at least partially through the activation of the AMPK/ATF3 pathway (114).

In an animal model study, ApoE^-/-^ mice were assigned to a control group, streptozotocin treated group or metformin treated group. Metformin treatment can reduce the metabolic disorder and AS caused by streptozotocin *via* reducing NLRP3 inflammasome activation as well as the disorder of thioredoxin-1/thioredoxin interacting protein. Metformin also inhibits ROS accumulation and NLRP3 inflammasome activation stimulated by high glucose in macrophages which is blocked by compound C. These results indicate that metformin can inhibit NLRP3 inflammasome activation in apoE^-/-^ mice and inhibit diabetes-accelerated AS, at least in part by activating AMPK and regulating thioredoxin-1/thioredoxin interaction protein (70).

In oxLDL-stimulated macrophages, metformin treatment can attenuate the protein expression and activation of the NLRP3 inflammasome. AMPK gene knockdown partially restores the activation of NLRP3 inflammasome, and PP2A inhibition restores the metformin-mediated down-regulation of NLRP3 and pre-IL-1β expression. Moreover, metformin-induced NF-κB inhibition also requires PP2A catalytic activity. These results show that metformin reduces NLRP3 protein expression and activation *via* AMPK and PP2A in oxLDL-induced macrophages (71). In RAW264.7 macrophages, LPS treatment significantly induced the expression of CXCL10 and CXCL11. Metformin can inhibit the phosphorylation of I-κBα and p65 by activating AMPK and prevent the stimulation of these chemokines, as well as IL-1 and IL-6 induced by LPS (53).

The heme produced by hemorrhage in the plaque may drive the protective M2-like phenotype through AMPK and ATF1. Metformin has a similar effect in macrophages to inhibit AS. In low-density lipoprotein receptor (Ldlr)^-/-^ hyperlipidemia mice, oral metformin inhibited the development of atherosclerotic lesions by activating AMPK and ATF1 in macrophages. Bone marrow transplantation experiments on AMPK knockout mice showed that the anti-atherosclerotic protection of metformin requires hematopoietic AMPK. The clinically relevant concentration (10μM) of metformin up-regulated LXRβ, Hmox1, ApoE, ABCA1, PDGF and IGF1, as well as increased several M2 markers and decreased iNOS in mouse BMDM by activating AMPK and ATF1. A similar effect was observed in human blood-derived macrophages. These results indicate that metformin can activate AMPK-ATF1-M2-like pathways in macrophages to inhibit AS in hyperlipidemia mice, and support the clinical study of metformin in people without DM but with high risk of AS (115, 116). In HFD-fed C57/6J mice, metformin treatment for 7 weeks decreased the blood levels of TNF-α and IL-6 as well as the expression of CD11c and MCP-1 (M1 macrophage markers) in AT. In palmitate-stimulated RAW264.7 cells, metformin decreased the secretion of IL-6 and TNF-α, while down-regulating M1 macrophages and up-regulating M2 macrophages. These findings show that metformin can regulate the polarization of macrophages to the anti-inflammatory M2 phenotype and improve low-grade inflammation in obesity by activating AMPK (101).

Acute AMPK activation enhances ischemic brain injury, but the clinical application metformin decreases the incidence of stroke (103). The duration of AMPK activation plays a crucial role for the effect of metformin on the prognosis of stroke (117). Mice received metformin treatment for 30 days after 24 hours of MCAO. Chronic metformin treatment after stroke significantly enhances brain AMPK activation, increases angiogenesis and neurogenesis, and makes microglia/macrophages tend to the M2 state in the ischemic brain (103). Advanced glycation end products (AGEs) are the main glucose-dependent inflammatory mediators of DM. In mouse macrophages, metformin pretreatment can inhibit the expression of cluster marker 86 (CD86) (M1 marker) induced by AGEs by activating AMPK, and promote the surface expression of CD206 (M2 marker) and IL- 10 mRNA expression (118).

In summary, AMPK activity is critical for the protective effects of metformin, including inhibiting monocyte cell migration and differentiation, inhibiting oxidative stress, inflammation and inflammasome activation, and macrophage polarization. The diseases and disorders involved include AS, stroke, diabetes, obesity and hyperlipidemia. These protective effects and mechanisms are occurring at least partially through the activation of AMPK.

### LXR/ABCG1

The initial and rate-limiting step of RCT is the HDL-mediated cholesterol efflux (88). ATP-binding cassette (ABC) cholesterol transporter-mediated cholesterol efflux from macrophages can reduce the progression of AS in patients (89). HDL glycosylation (3-DG incubation) obviously decreased HDL-mediated cholesterol efflux from MDM. Glycated HDL particles cannot effectively act as ABCG1-mediated cholesterol efflux receptors, which can partially explain the acceleration of AS in DM patients, and metformin treatment restores HDL-mediated cholesterol efflux (88). Similarly, RAW264.7 cells were stimulated by oxLDL (50μg/ml) for 24 h, and then treated with metformin (15μM) for 24 h. Metformin promotes the outflow of cholesterol to HDL by increasing ABCG1 expression, which decreases foam cell formation induced by oxLDL. At the same time, metformin increases the IL-10 secretion that is impaired by oxLDL, which is an important anti-inflammatory cytokine in AS (89). In HFD-fed apoE^-/-^ mice and macrophages, metformin treatment also showed significant RCT. Mechanism studies have shown that AMPK activates HDL in mice, with higher paraoxonase 1 activity, lower myeloperoxidase activity and lower HDL inflammation index. Metformin also up-regulated the expression of ABCG1 and ABCA1 in macrophages, as well as lecithin:cholesterol acyltransferase (LCAT) and SR-B1 in the liver (104).

### NF-κB

Numerous studies have shown that metformin can block the NF-κB signaling pathway in macrophages. For example, in BMDM, metformin treatment attenuated the LPS-stimulated phosphorylation of p65 and JNK1 and the up-regulation of pro-inflammatory cytokine levels (119). The anti-atherosclerotic effect of macrophage-derived apoE is well known. Metformin (1-3 mM) reversed the reduction of apoE expression in macrophages induced by LPS *via* reducing the nuclear translocation of NF-κB (120). In human SMCs, macrophages and ECs, metformin can dose-dependently inhibit the release of the IL-6 and IL-8 stimulated by IL-1β in these three cell types. Mechanism studies have indicated that metformin can block NF-κB nuclear translocation by inhibiting the PI3K-Akt pathway (54). In oxLDL-stimulated macrophages, NF-κB inhibition induced by metformin requires PP2A catalytic activity (71). Metformin may not require AMPK to inhibit NF-κB activation. For example, in AMPK null fibroblasts and macrophages, metformin inhibits the NF-κB signaling pathway induced by LPS (121). The anti-inflammatory role of metformin may partially contribute to the effect of metformin in reducing MACE independently of its hypoglycemic effect (54).

### Sirt1

The ARDS model was established by injecting LPS into mice treated with metformin in advance and mice in the control group. ARDS model mice developed neutrophil accumulation, vascular exudation and pulmonary edema, and the expression of IL-1β, TNF-α, IL-6 and IL-17 were up-regulated. Metformin treatment partially reversed indicators of pulmonary arterial damage and reduced LPS-induced deaths. The expression of SIRT1 in metformin-treated alveolar macrophages (NR8383) was higher than that of LPS alone, while the levels of p-p38, p-ERK and p-NF-κB were significantly down-regulated. Further research showed that metformin decreased miR-138-5p expression, while miR-138-5p could target SIRT1 and inhibit its expression. These results indicate that metformin increases SIRT1 expression and inhibits the MAPK pathway by reducing the expression of mir-138-5p, thereby reducing the lung injury and the expression of inflammatory factors caused by LPS (58).

### ABCG5/8

ATP-binding cassette transporter G5 and G8 (ABCG5/8) mediates the final step of RCT, which promotes the hepatobiliary transport of cholesterol. In human or mice primary hepatocytes, metformin treatment increased ABCG5/8 expression. Recent studies have shown that metformin may increase the RCT of macrophages. In HFD-fed mice, metformin treatment reduced the occupation of the transcription repressor in Abcg5/8 and significantly increased Abcg5/8 expression. These findings support that metformin may protect the cardiovascular system *via* up-regulating the reverse transport of cholesterol (131).

### HMGB1

High mobility group box-1 (HMGB1) protein was identified in 1973 and can be released from various cells. It is a typical damage-related molecular model protein (122, 123). Several modifications of HMGB1 facilitate its transport to the cytoplasm and release from the cell either actively or passively. When released outside the cell, HMGB1 is essential for inflammation. Its biological function is through interaction with receptors, such as receptor for advanced glycation end products (RAGE) (122, 123, 132). HMGB1 is released by necrotic cells and leads to inflammatory responses through its cytokine-like activity; thus, HMGB1 is a potential target for the development of anti-inflammatory therapies (133). By using a biotinylated metformin analog for affinity purification, it was found that metformin directly binds to the C-terminal acid tail of HMGB1. Moreover, in the paracetamol-stimulated acute liver damage model, HMGB1 released from damaged cells exacerbated liver injury, and metformin treatment inhibited the liver damage (133). In addition, the exaggerated release of NETs, reduced NET clearance as well as impaired clearance of apoptotic cells may induce persistent inflammation of ARDS. Neutrophils from ARDS patients showed a decrease in apoptosis and an increase in NETs formation. Incubation of neutrophils with bronchoalveolar lavage (BAL) fluid of ARDS can promote NETs formation. In ARDS patients, macrophage phagocytosis of NETs and apoptotic neutrophils is decreased. Neutralization of HMGB1 antibody in BAL fluid or activation of AMPK in macrophages in BAL fluid improved cell swelling and NETs clearance. These results suggest that using metformin to restore AMPK activity or specifically neutralize HMGB1 in BAL fluid may reduce persistent lung inflammation during ARDS (134). Similarly, metformin can significantly reduce the LPS-stimulated macrophage inflammatory response *in vivo* (mice) and *in vitro* (RAW 264.7 cells) by inhibiting HMGB1 secretion, and improve the survival rate of endotoxemic mice (135).

### FOXO1/FABP4

Lipid accumulation in macrophages induces the development of AS. In addition to reducing lipid accumulation in adipocytes, metformin also reduces palmitic acid-stimulated lipid accumulation in macrophages. Mechanistic studies show that metformin reduces FABP4 expression and promotes ocarnitine palmitoyltransferase I (CPT-1) expression, which is related to palmitic acid-stimulated lipid accumulation in macrophages. Further studies show that FOXO1 siRNA inhibits FOXO1 expression and significantly reduces basal and palmitic acid-induced FABP4 expression, while metformin reduces FABP4 expression *via* reducing nuclear translocation of FOXO1. These findings indicate that metformin decreases lipid accumulation *via* inhibiting FOXO1-induced FABP4 transcription in macrophages (91).

### The Mechanism of Metformin Actions Independent of AMPK

AMPK consists of a catalytic subunit (α) and regulatory subunits (β and γ) (136, 137). The α subunit of AMPK has two subtypes, AMPKα_1_ and AMPKα_2_, which differentially exist in different tissues (138). Among them, the AMPKα_1_ mainly exists in SMCs (139), macrophages (140) and AT (141), while AMPKα_2_ expression is much higher in cardiomyocytes (142). AMPK exhibits a very important regulatory function in both metabolism and CVD (143).

AMPK α_2_ knockout $\left(AMPK\alpha_{2}^{-/-}\right)$ mice have high ER stress and atherosclerotic phenotypes of aortic ECs (144). The genetic defect of AMPKα_1_ (but not AMPKα_2_) enhances vascular calcification. Long-term administration of metformin to activate AMPK can reduce vascular calcification in ApoE^-/-^ mice, but not in ApoE^-/-^/AMPKα1^-/-^ mice. These studies reveal that both AMPKα_1_ and AMPKα_2_ exhibit a very important effect in AS, and metformin inhibits arteriosclerosis calcification mainly by activating AMPKα_1_ (39). As we mentioned earlier, metformin plays a regulatory role in macrophage oxidative stress, inflammation, macrophage polarization, foam cell formation, and apoptosis. However, the specific mechanisms are still not clear. BM transplantation experiments on AMPK knockout mice showed that the anti-atherosclerotic protection of metformin requires hematopoietic AMPK, which suggests that AMPK occupies a crucial position in the modulation of macrophage function by metformin (115). However, in AMPK null fibroblasts and macrophages, metformin blocked LPS-stimulated NF-κB signaling pathway (including preventing the nuclear translocation of NF-κB, and inhibiting IκB and IKKα/β phosphorylation) (121). The differences in the mechanism of metformin *in vivo* and *in vitro* need to be further studied. It may be impractical to perform macrophage-specific AMPK knockout *in vivo*, because MDM are an important link in the development of AS.

In the signaling of the regulation of liver glycogen homeostasis, metformin inhibits complex I and prevents the production of mitochondrial ATP, thereby increasing the ratio of cytoplasmic ADP: ATP and AMP: ATP, which leads to AMPK activation (145). However, the up-regulation of AMP: ATP also inhibits fructose 1, 6-bisphosphatase (FBPase), leading to an acute reduction of gluconeogenesis (146), and at the same time inhibited adenylate cyclase and reduced the production of cAMP (147). Acute treatment with AICAR or metformin can also inhibit glucose production in hepatocytes of wild type mice or mice lacking both AMPKα_1_ and AMPKα_2_ in the liver, and metformin can improve glucose tolerance in the two mouse strains (148). This indicates that the acute inhibitory role of metformin on the production of hepatic glucose is independent of AMPK, and hence that AMP inhibition of fructose-1,6-bisphosphatase is a possible explanation. However, the main long-term hypoglycemic role of metformin is to increase hepatic insulin sensitivity, which is mediated by AMPK in mouse experiments. These results indicate that the acute and chronic pharmacological mechanism of action of metformin is both AMPK-dependent and independent. Therefore, we speculate that the AMPK-independent effect of metformin in macrophages (such as the metformin inhibition of LPS-induced NF-κB signaling pathway) may be an acute effect.

**Figure 3:**
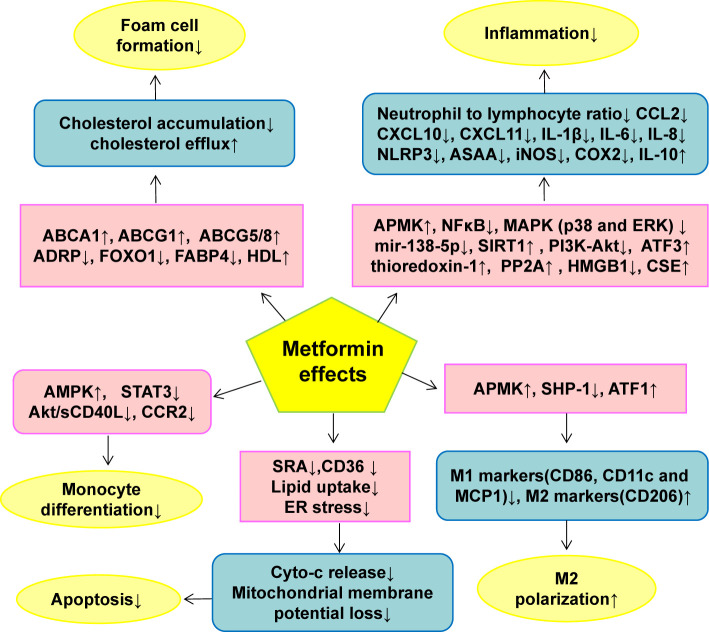
The effect of metformin on monocyte/macrophage functions in AS. Metformin inhibits monocyte/macrophage dysfunction *via* modulating the expression and activity of genes or proteins closely related to monocyte differentiation, macrophage inflammation, M1/M2 polarization, foam cell formation and macrophage apoptosis. ↑indicates increase or activation, and ↓indicates decrease or suppression. ABCA1, ATP-binding cassette transporter A1; ABCG1, ATP-binding cassette transporter G1; ABCG5/8, ATP-binding cassette transporter 5/8; ADRP, adipogenic differentiation-associated protein; Akt, protein kinase B; AMPK, 5’-adenosine monophosphate-activated protein kinase; ASAA, acute-phase serum amyloid A; ATF, Activation of transcription factor; CCL2, C-C motif chemokine ligand 2; CCR2, CC chemokine receptor 2; CD, cluster of differentiation; CXCL, Chemokine C-X-C ligand; CSE, Cystathionine γ-lyase; cyto-c, cytochrome c; ER, endoplasmic reticulum; ERK, Extracellular signal-regulated kinase; FABP4, Fatty acid binding protein 4; FOXO1, Forkhead box transcription factor O1; HDL, high-density lipoprotein; HMGB1, high mobility group box-1; iNOS, inducible nitric oxide synthase; IL, Interleukin; MAPK, mitogen-activated protein kinase; MCP1, Monocyte chemoattractant protein 1; NF-κB, nuclear factor kappa B; NLRP3, NOD-like receptor family pyrin domain containing 3; PI3K, phosphatidylinositol 3-kinase; PP2A, protein phosphatase 2A; sCD40L soluble CD40 ligand; SH2, Src homology 2; domain-containing protein tyrosine phosphatase 1(SHP-1), SIRT1, Sirtuin-1; SRA, scavenger receptor class A; STAT3, Signal transducer and activator of transcription 3.

Metformin reduces macrophage oxidative stress and inflammatory cytokine production by AMPK activation, but the residual effect of metformin remains significant even after compound C treatment (79). Although compound C has AMPK off-target effects, the study also shows that metformin may not completely rely on AMPK to inhibit macrophage inflammation. In addition, in AMPK null macrophages, metformin inhibited the NF-κB signaling pathway induced by LPS. Therefore, metformin’s inhibition of macrophage inflammation may partly directly depend on NF-κB. The molecular targets of metformin in ameliorating macrophage dysfunction was summarized in **Figure 3**.

## Current Perspectives and Limitations

### Research on Non-Coding RNA (Including miRNA and lncRNA) Mechanisms in the Pharmacological Actions of Metformin in AS

Recent evidence has shown that AS is a disease with epigenetic components. Various documented epigenetic modifications (including histone methylation/acetylation and non-coding RNA) exhibit an important regulatory effect in all aspects of the pathogenesis of AS, including EC damage and dysfunction, SMC proliferation, migration and phenotypic modulation, monocyte differentiation, macrophage-mediated inflammation and polarization and foam cell formation, as well as platelet aggregation. Several small-molecule epigenetic drugs (including histone deacetylase inhibitors, trichostatin A (TsA) and suberoylanilide hydroxamic acid (SAHA) as well as DNA methyltransferases inhibitors 5-Azacytidine and 5-aza-2’-deoxycytidine) approved by FDA have shown promise in preclinical studies for the treatment of AS (149).

Monocyte/macrophages can develop long-term pro-inflammatory and pro-atherosclerotic phenotypes after treatment with inflammatory stimuli (including oxLDL). This inherent immune memory is produced due to changes in metabolic pathways and epigenetic reprogramming of histone modifications. The persistence of blood hyperresponsive monocytes in the body may be due to training in myeloid progenitor cells in BM. In patients with AS and those with dyslipidemia, there are well-trained immunophenotypic monocytes (150). These findings indicate that monocyte-transformed macrophages have amazing inflammatory plasticity, allowing chronic inflammation and atherosclerotic plaque to persist (151).

Non-coding RNA (ncRNA) include microRNA (miRNA), long non-coding RNA (lncRNA) as well as circular RNA (circRNA) (152). ncRNA can regulate the occurrence and development of AS by interacting with proteins, DNA and RNA (153). Inhibiting the role of macrophages in inflammation is crucial for inducing the regression of AS, and miRNA has emerged as a key regulator of macrophage phenotype. MiRNA which is a small non-coding RNA that modulates gene expression, is very stable in the circulation and therefore has the potential as a therapeutic agent and/or biomarker in the case of AS. miRNA (such as miR-155) is dysregulated during the development of AS and is an important mediator of macrophage inflammation and polarization. Inhibiting macrophage-specific miR-155 can reduce atherosclerotic inflammation (154). Conjugated linoleic acid (CLA), a peroxisome proliferator-activated receptor (PPAR)-γ agonist, reduces inflammation of monocytes and macrophages through regulation of miRNA, and exerts anti-atherosclerosis effect through this mechanism (154). In LPS-stimulated macrophages, metformin exhibits an anti-inflammatory role (decrease the expression of IL-6 and TNFα) on macrophages by inducing Dicer (a key miRNA biogenesis enzyme) and then up-regulating miR-125b-5p and miR-34a-5p (155).

lncRNA is a non-protein coding RNA group whose length are more than 200 nucleotides. lncRNA regulates the complex gene regulatory network in macrophages to participate in the process of AS (156–158). Many lncRNAs are involved in macrophage cholesterol deposition, inflammation and foam cell formation, such as LASER, LeXis and CHROME. MANTIS, lncRNA-CCL2 and MALAT1 are associated with vascular inflammation. In addition, some lncRNAs are closely related to the response to statin therapy, such as NEXN-AS1 or LASER. Some lncRNAs can also serve as biomarkers of CVD (159). LncRNA is a potential new therapeutic target for CVD, but the research on lncRNA in AS is still in its infancy (159).

NcRNA, including miRNA and lncRNA, exhibit an important effect in modulating the phenotype and function of macrophages through epigenetic modification of macrophages. At present, whether metformin affects the function of macrophages by acting on miRNA or lncRNA is still lacking, which may be an important direction for future research.

### Single Cell Sequencing Plays a Very Important Role in Revealing the Pathogenesis of AS

Traditional genetic information research methods are carried out at the multicellular level. Therefore, the final signal value is actually the average of multiple cells, and the information of heterogeneity is lost. Single-cell sequencing can detect heterogeneous information that cannot be obtained by sequencing mixed samples (160–162). Currently, barcode-based single-cell identification/combinatorial indexing is often adopted, and the information of hundreds of single cells can be measured by building a database at a time (163, 164). In recent years, single-cell sequencing has exerted an important effect in revealing the pathogenesis of diseases and targets for drug therapy.

Atherosclerotic plaque contains autoantibodies, and there is a link between AS and autoimmunity (165–167). Through the high-throughput single-cell analysis of AS-related antibody library, including antibody gene sequencing for more than 1,700 B lymphocytes in control mice and atherosclerotic Ldlr^-/-^ mice, 56 antibodies were identified. In-depth proteomic analysis determined that ALDH4A1 is the target antigen for one of the above autoantibodies, A12. The circulating ALDH4A1 is increased in humans and mice with AS. Moreover, in Ldlr^-/-^ mice, infusion of A12 antibody can delay atherosclerotic plaque formation and reduce blood LDL and free cholesterol, which indicates that The ALDH4A1 antibody can prevent the development of AS and may have therapeutic potential in CVD (168).

Using single-cell RNA sequencing, Wirka, RC et al. characterized the transcriptome phenotype of SMCs regulated in atherosclerotic lesions of human and mice arteries, and found that these SMCs transformed into unique fibroblast-like cells, called “fibromyocytes”, rather than the classic macrophage phenotype. The specific knockout of TCF21 (a basic helix-loop-helix transcription factor) inhibits the phenotypic regulation of SMC in mice, resulting in fewer fibromyocytes in the lesions. In addition, TCF21 expression is strongly correlated with SMC phenotype regulation in diseased human coronary arteries, and higher expression of TCF21 is correlated with reduced CAD risk. These results show the protective effect of TCF21 and SMC phenotypic regulation in CAD (169).

The above two studies show the important mechanism by which B lymphocytes and SMCs play a role in the progress of AS through single cell sequencing. In future research, this technology should be used to analyze the plaques of patients with AS combined with CVD and treated with metformin and we look forward to discovering specific molecular targets and sensitive cell communities for macrophages.

### Neutrophil Extracellular Traps (NETs) May Play an Important Role in the Anti-Atherosclerotic Effects of Metformin

The most abundant blood white blood cell of healthy humans is neutrophils (170). Neutrophils exert a very important effect in innate immune defense (171). NETs are reticular fiber structures consisted of DNA-histone complexes and proteins produced by activated neutrophils (172). NETs were discovered ten years ago as part of the first host defense system against invading microorganisms (173). Accumulating evidence indicates that NETs are also present in malignant tumors, AS and autoimmune diseases, including systemic lupus erythematosus (SLE), rheumatoid arthritis (RA), gout and psoriasis. Recent evidence indicates that NETs are contributors to venous and arterial thrombosis (174). The imbalance between NETosis (the process of NETs formation) and NETs degradation may be related to autoimmune diseases (175). NETs are found in atherosclerotic lesions in human and animal models, and NETs are involved in multiple components of atherogenesis. For example, NETs induce endothelial cell dysfunction and apoptosis, and promote the production of autoantibodies against double-stranded DNA. NETs may also play a role in promoting thrombosis because they form a fibrin-like matrix where platelets adhere, become activated and aggregate. NETs induce oxidative stress leading to oxidization of HDL particles, thereby reducing their beneficial cholesterol efflux ability (176). In addition, NETs contain myeloperoxidase, which has the potential to modulate LDL oxidation (177, 178). In humans, inflammasome activation and NETosis may result in atherosclerotic plaque erosion and thrombosis, especially in patients with T2DM, clonal hematopoiesis or chronic kidney disease (179). In patients with diabetes, the *in vitro* studies show that induction of NETosis and the circulating concentration of NETs-related proteins appear to increase. NETosis seems to be part of the abnormal response to diabetes damage, which, in turn, can promote or aggravate end-organ complications (180).

NETs have been shown to exert an important effect in the pathogenesis of SLE *via* inducing activation of plasmacytoid dendritic cells (PDCs) and type I interferon (IFN) pathways (181). The incidence of MACE and subclinical AS is increased in SLE patients (182). NETs can destroy and kill ECs, and increase inflammation in atherosclerotic plaques, and this may accelerate AS in SLE patients. Anti-IFN-α therapy and other new drugs (including peptidylarginine deiminase inhibitor 4, N-acetylcysteine and DNase I) can target NETs and have potential in the therapy of autoimmune diseases (183). Metformin treatment promotes the differentiation of T cells into memory and regulatory T cells and reduces the ability of neutrophils to engage in NETosis. Because metformin has an inhibitory effect on the pro-inflammatory phenotype of immune cells, it has shown beneficial results in animal models of autoimmune diseases as well as in some relevant clinical trials (184). For example, additional treatment of mild and moderate SLE with metformin reduced clinical flares, body weight and prednisone exposure. Further studies have shown that metformin reduces PMA-induced neutrophil NETs formation and CpG-induced PDC-IFNα production, indicating that metformin may be an adjuvant therapy for SLE (185). The formation of NETs in ARDS patients is significantly enhanced. Incubation of neutrophils to BAL fluid of ARDS can promote NETs formation. In ARDS patients, macrophages phagocytosis of apoptotic neutrophils and NETs is reduced. The activation of AMPK in macrophages in BAL fluid improves cell swelling and NETs clearance. These results indicate that the administration of metformin to restore AMPK activity may reduce persistent lung inflammation during ARDS by improving macrophage function and NETs clearance (134).

In summary, NETs not only promote the occurrence and development of SLE and RA, but also play a very important role in diabetes and AS. This suggests that the study of the effect of metformin on NETs is an important area of research to broaden the understanding of the full clinical potential of metformin.

### Combination Therapy Is an Important Research Area for Metformin Treatment

Metformin, as an oral biguanide hypoglycemic drug, is mainly used in the clinical therapy of T2DM and it has efficacy and safety as mono- and combination therapy with other anti-hyperglycemic medications (186, 187). In addition to its protective role in diabetes-associated CVD and potentially CVD occurring in patients without diabetes, multiple other therapeutic effects of metformin have been recognized, including reducing the risk of dementia (improving cognitive impairment) (188), weight loss (189), anti-aging (190, 191), suppressing air pollution damage (inhibiting inflammation and thrombosis) (107), improving PCOS (192, 193), and reversing lung fibers (194), inhibiting hair loss (195), reducing the risk of death from kidney failure and kidney disease (196, 197), and preventing and treating some cancers (198–201). Macrophages are distributed in the circulation and tissues and aggregate under a variety of pathological conditions and play an important role in many pathological processes by regulating inflammation, such as cardiovascular disease (202), obesity (203), T2DM (203), T2DM (203), cancer (204), aging (205) and dementia (206). Therefore, we speculate that improving the function of macrophages is the cellular basis for the pleiotropic potential of metformin. To expand on this correlation, below we focus on the potential of metformin in combination with drugs that improve macrophage function in CVD, including hypoglycemic agent-sodium glucose cotransporter 2 inhibitors (SGLT2i), lipid-lowering drug (statins) and an anti-inflammatory drug (IL-1β inhibitor).

SGLT2i, including empagliflozin, dapagliflozin and canagliflozin, have been shown recently to reduce the hospitalization rate of patients with heart failure and decrease the mortality rate from cardiovascular diseases (207–210). Inhibiting the inflammatory actions of macrophages is one of the important mechanisms through which SGLT2is exert CVD protection. For example, subjects with T2DM at high cardiovascular risk were given sulfonylurea or empagliflozin (SGLT2i) treatment for 30 days, and the activation of NLRP3 inflammasome in macrophages was analyzed. Although the hypoglycemic ability of SGLT2i is close to that of sulfonylurea, it has greater decrease of IL-1β production, accompanied with decreasing blood insulin levels and increasing blood β-hydroxybutyrate (BHB). *In vitro* experiments with macrophages supported the effect of low insulin and increased BHB levels on the inactivation of NLRP3 inflammasome. SGLT2i attenuates the activation of NLRP3 inflammasome, which might underlie its cardiovascular protective effect (211). In addition, in a study in which C57BL/6J mice were fed with HFD or HFD plus empagliflozin for 16 weeks empagliflozin inhibited HFD-caused liver steatosis, insulin resistance and weight gain. In addition, empagliflozin decreases the accumulation of M1 macrophages, simultaneously induces the anti-inflammatory M2 macrophage phenotype in liver and white AT, and reduces obesity-related chronic inflammation (such as blood TNFα level). Therefore, empagliflozin enhances fat utilization, inhibits insulin resistance as well as reducing obesity-induced inflammation *via* polarizing M2 macrophages in liver and white AT, thereby inhibiting weight gain (212). Critically, in patients with diabetes and the associated increased cardiovascular risk, metformin combined with SGLT2i (empagliflozin) as well as specific GLP-1 receptor agonists (semaglutide and liraglutide) reduced all-cause mortality and cardiovascular death (187).

In addition, compared with metformin monotherapy, atorvastatin and metformin co-treatment reduced the level of TNF-α after oral glucose in patients with T2DM, and partially prevented the increase in blood glucose caused by glucose load (213). The combination of colesevelam hydrochloride and metformin, a bile acid chelator, further improved the blood glucose profile and lipid indicators [fasting blood glucose, fructosamine, total cholesterol, LDL-c, high-sensitivity C-reactive protein (hsCRP)] in T2DM patients (214). Anti-inflammatory drugs (such as canakinumab) have benefits in the treatment of AS. For example, in 10,061 patients with previous myocardial infarction and elevated CRP levels, IL-1β-targeted canakinumab (150 mg, every 3 months) treatment (median follow-up time of 3.7 years) significantly reduced recurrence cardiovascular events (HR=0.85) (including non-fatal stroke, non-fatal myocardial infarction, or cardiovascular death), and this action was independent of changes in blood lipid profiles (215). Thus, the combination of metformin and canakinumab may have a better role in the prevention and treatment of CVD.

## Safety and Side Effects

**Figure 4:**
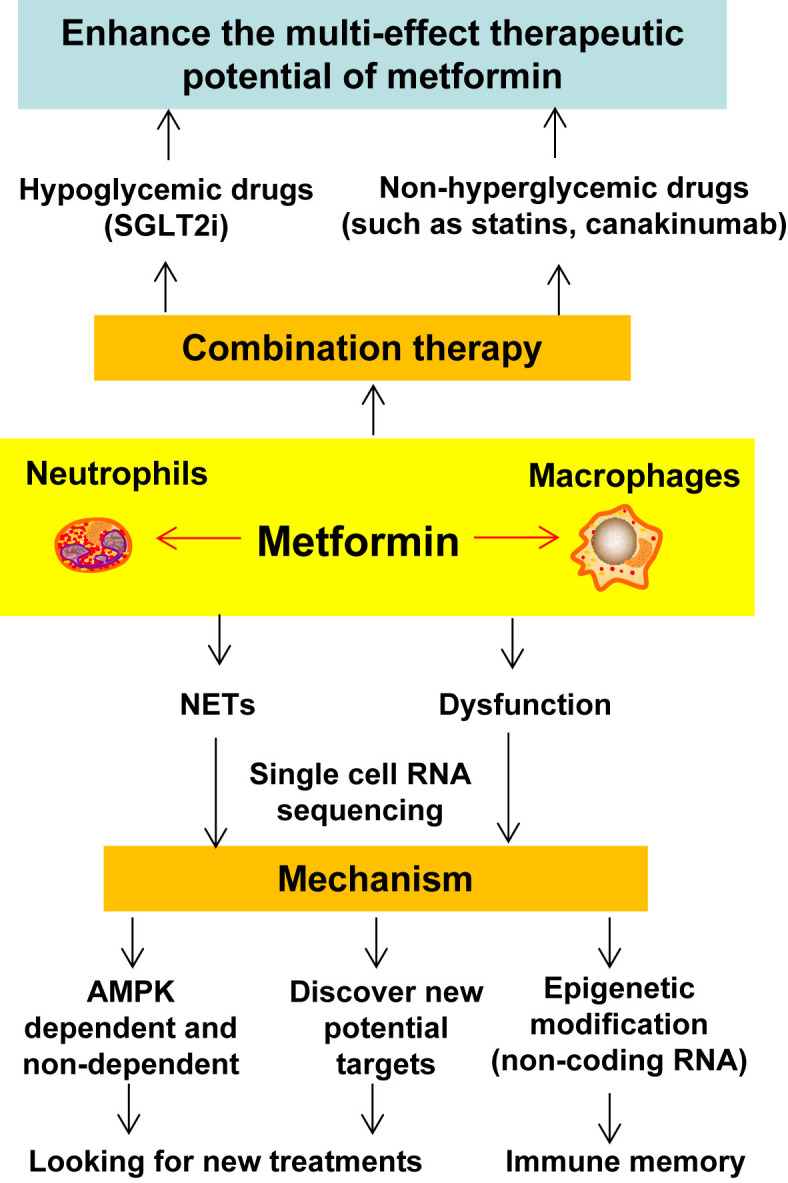
Future directions of studying the pharmacological actions and molecular mechanisms of metformin in atherosclerosis. Numerous studies have demonstrated the protective effects of metformin on macrophage function, however, the effect of metformin on atherosclerosis in experimental animal models and humans are warranted. Recent studies have shown that neutrophils (the most abundant white blood cells in healthy people) can participate in the development of AS through the formation of NETs. Moreover, single-cell RNA sequencing plays an increasingly important role in revealing various physiological and pathological mechanisms, and can be further utilized to uncover the effects of metformin on monocytes/macrophages and neutrophils. This will provide important clues for the study of immune memory and the exploration of new treatment methods. In addition, the combination of metformin and other drugs (such as statins and SGLT2i) synergistically improves the function of macrophages, further enhancing the pleiotropic effects of metformin. NETs, neutrophil extracellular traps; SGLT2i, sodium-glucose cotransporter 2 inhibitors.

Metformin has been used to treat diabetes for more than 60 years (20).With its safety profile, effectiveness and significant low cost advantage, metformin is the first-line treatment for most T2DM patients (20). The common gastrointestinal side effects of metformin are usually mild and transient. Lactic acidosis is the only serious adverse reaction, but the incidence is very low (9 times per 100,000 people per year) (216, 217). New drugs, including SGLT-2 inhibitors and GLP-1 receptor agonists, also show significant cardiovascular benefits, but the long-term safety of these new drugs needs to be confirmed (218). Compared with other hypoglycemic drugs, the clinical experience and safety data of metformin are still significantly better (219, 220). For the common side effects of metformin, it is possible to use sustained-release preparations, controlled (late) release products and to control the dose of metformin and optimally mange patients and minimize adverse effects (20). Future prospects of metformin in atherosclerosis was summarized in **Figure 4**.

## Conclusion

Metformin has evolved as the first-line hypoglycemic drug for the treatment of diabetes and extensive laboratory and clinical research has revealed that it has the potential to treat many other diseases (such as CVD, cancer and aging). Macrophages, which are distributed in the circulation and tissues and aggregate under a variety of pathological conditions, can play an important role in a variety of diseases by regulating inflammation. Considerable evidence indicates that metformin can improve the dysfunction of macrophages which is a cause of AS. We speculate that improving the function of macrophages may be the basis for the expanding therapeutic potential of metformin. Combined with other drugs that improve the function of macrophages (such as SGLT2i, statins and IL-β inhibitor), this may help to further strengthen the pleiotropic actions and thus the therapeutic potential of metformin. In addition, there is evidence that metformin can inhibit the formation of NETs, which may be related to the effect of metformin on improving macrophage function. In terms of research depth, single-cell sequencing helps to further clarify the mechanism of metformin and help to discover new targets for improving the function of macrophages and controlling or reducing the role of these cells in multiple disease processes and states.

## Author Contributions

SX, LT, and JW conceived the manuscript. XF, and WC wrote the manuscript and designed the figures. XF, WC, XN, SX, LT and JW edited the manuscript. PL edited the manuscript while providing input to aspects of diabetes, atherosclerosis and CVD. All authors contributed to the article and approved the submitted version.

## Funding

This study was supported by grants from National Natural Science Foundation of China [Grant Nos. 81941022 to JW, 81530025 to JW, 81773955 to LT, 82070464 to SX, 82003740 to XF]. This work was also supported by Strategic Priority Research Program of Chinese Academy of Sciences [Grant No. XDB38010100 to JW], Program for Innovative Research Team of the First Affiliated Hospital of USTC (to JW), and the National Key R&D Program of China [Grant No. 2017YFC1309603 to JW]. This work was also supported by Local Innovative and Research Teams Project of Guangdong Pearl River Talents Program [2017BT01S131], and the Fundamental Research Funds for the Central Universities (WK9110000079 to XF).

## Conflict of Interest

The authors declare that the research was conducted in the absence of any commercial or financial relationships that could be construed as a potential conflict of interest.
